# Ethynyl-substituted benzosiloxaboroles: the role of C(π)⋯B interactions in their crystal packing and use in Cu(i)-catalyzed 1,3-dipolar cycloaddition†

**DOI:** 10.1039/d4ra02137a

**Published:** 2024-05-17

**Authors:** P. Pacholak, K. Durka, K. Woźniak, J. Krajewska, A. E. Laudy, S. Luliński

**Affiliations:** a Faculty of Chemistry, Warsaw University of Technology Noakowskiego 3 00-664 Warsaw Poland krzyysztof.durka@pw.edu.pl sergiusz.lulinski@pw.edu.pl; b University of Warsaw, Faculty of Chemistry Pasteura 1 02-093 Warsaw Poland; c Department of Pharmaceutical Microbiology and Bioanalysis, Medical University of Warsaw Banacha 1b 02-097 Warsaw Poland alaudy@wp.pl

## Abstract

The synthesis and characterization of two novel 6-ethynyl-7-halogen substituted benzosiloxaboroles (Hal = F, Cl) is reported. The crystal structures of these compounds show a unique type of supramolecular assembly dictated by distinctive C(π)⋯B interactions resulting in the formation of columnar networks involving alternating ethynyl groups and boron atoms. The QTAIM, NBO and NCI analyses were performed in order to obtain a deeper quantitative insight into the nature of these interactions including energy and charge density distribution. The fluoro derivative 1c was used as a starting material in Cu-catalyzed 1,3-dipolar cycloaddition reactions with substituted benzenesulfonyl azides giving rise to benzosiloxaboroles with pendant 1-(arylsulfonyl)-1,2,3-triazole-4-yl functionalities or analogous ionic species, *i.e.*, 1,2,3-triazolium arylsulfonates. Screening of antimicrobial activity of obtained derivatives against a wide selection of Gram-positive and Gram-negative bacteria as well as fungi strains was performed and the obtained results were compared with the data obtained previously for related benzosiloxaborole derivatives.

## Abstract 2

Ethynyl-substituted benzosiloxaboroles feature distinctive C(π)⋯B interactions in the crystalline state and are useful in Cu(i)-catalyzed 1,3-dipolar cycloaddition reactions.

## Graphical abstract

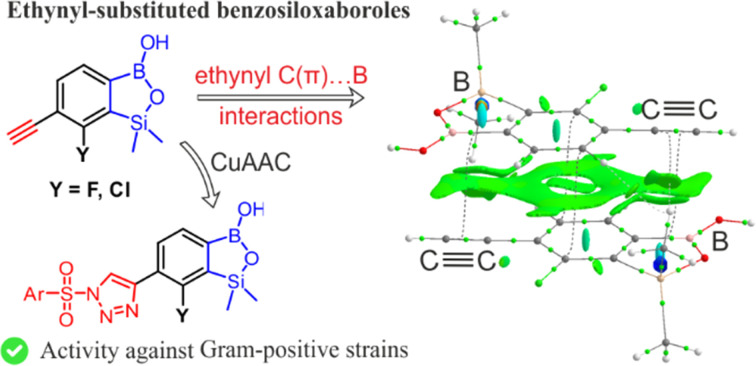

## 1 Introduction

A specific class of cyclic boron hemiesters called benzoxaboroles have been known for 70 years^1^ but they have attracted increased attention only during the last 15 years in the field of medicinal chemistry.^2^ Extensive studies revealed that functionalized benzoxaboroles are promising small-molecule therapeutic agents possessing antibacterial, antifungal, anti-inflammatory and anticancer activity.^3^ For example, fluorinated benzoxaborole called tavaborole (trade name Kerydin) exhibits high antifungal activity and was commercialized to treat onychomycosis – a fungal infection of the nail and nail bed.^4^ 5-(4-Cyanophenoxy)benzoxaborole called crisaborole (trade name Eucrisa) was approved for the treatment of mild-to-moderate atopic dermatitis (eczema).^5^ The activity of benzoxaboroles relies primarily on the binding of the Lewis acid boron centre to the biological target *via* the formation of a strong covalent bond. The mechanism of action of benzoxaboroles relies on their physicochemical specificity based on the enhanced character of the boron atom. The examples of approved benzoxaborole therapeutic agents as well as other derivatives which are currently under investigation demonstrate that the mode of bioactivity depends on structural modification of the benzoxaborole core. In 2015, our group proposed benzosiloxaboroles as silicon congeners of benzoxaboroles and demonstrated high antifungal activity of simple fluorinated derivatives.^6^ Further research revealed the potency of selected derivatives including oxaborole–benzosiloxaborole hybrid II as KPC β-lactamase inhibitors.^7^ Recently, we found that the antibacterial activity of benzosiloxaboroles involves a different mechanism than for related benzoxaboroles pointing to the importance of the introduced structural change.^8^ In fact, replacement of the CH_2_ with the SiMe_2_ group in the oxaborole ring results in increased Lewis acidity and lipophilicity which may be responsible for specific biological activity. Recently, we have also studied crystal structures of functionalized benzosiloxaboroles showing that there is a strong effect of substitution pattern on their supramolecular assembly, mostly manifested by formation of various hydrogen-bonded motifs.^9^ Those results revealed that benzosiloxaboroles are interesting also from the perspective of crystal engineering.

In medicinal chemistry, the conjugation a core of a molecule with different pharmacophores is one of key synthetic concepts as resulting hybrid structures may show higher affinity to a biological target. Herein, we turned our attention to triazole scaffold as it is present in many compounds showing diverse biological properties. For example, vicinal diaryl triazoles were found to be promising tubulin polymerization inhibitors, COX-2 inhibitors, and CB1 receptor antagonists.^10^ Essramycin (Scheme 2), is (1,2,4-triazolo)pyrimidine derivative active against several Gram-positive and Gram-negative bacteria such as *Bacillus subtilis*, *Staphylococcus aureus*, *Micrococcus luteus*, *E. coli*, and *Pseudomonas aeruginosa*.^11^ Triazole-containing compounds are widely used as topical and systemic antifungal agents. Fluconazole, the first-generation 1,2,4-triazole antifungal agent, is used for fungal infections, including candidiasis, blastomycosis, cryptococcosis, histoplasmosis, coccidioidomycosis, dermatophyteosis and pityriasis versicolor.^12^ 1,2,3-Triazole moiety is a part of tazobactam – inhibitor of β-lactamases from class A according to Ambler classification (especially SHV, TEM and CTX-M enzymes).^13^ Tazobactam combined with the β-lactam antibiotic such as piperacillin is used to treat infections caused by *Pseudomonas aeruginosa*. Moreover, tazobactam with the new cephalosporin ceftolozane is dedicated for treatment of the extended-spectrum β-lactamase (ESBL)-producing *Enterobacterales* and multidrug-resistant (MDR) *P. aeruginosa* infections.^13*b*^ Rufinamide is a new anti-epileptic drug used in combination with other medication and therapy to treat Lennox–Gastaut syndrome and various other seizure disorders.^14^ In the context of this work, a recent synthesis of a novel boronic acid (MB076) with a pendant 1,2,3-triazole-4-carboxylic acid moiety as a highly effective inhibitor of class C *Acinetobacter*-derived cephalosporinases is interesting.^15^ 6-Substituted triazolyl benzoxaboroles were very recently identified as selective carbonic anhydrase inhibitors.^16^

**Scheme 1:**
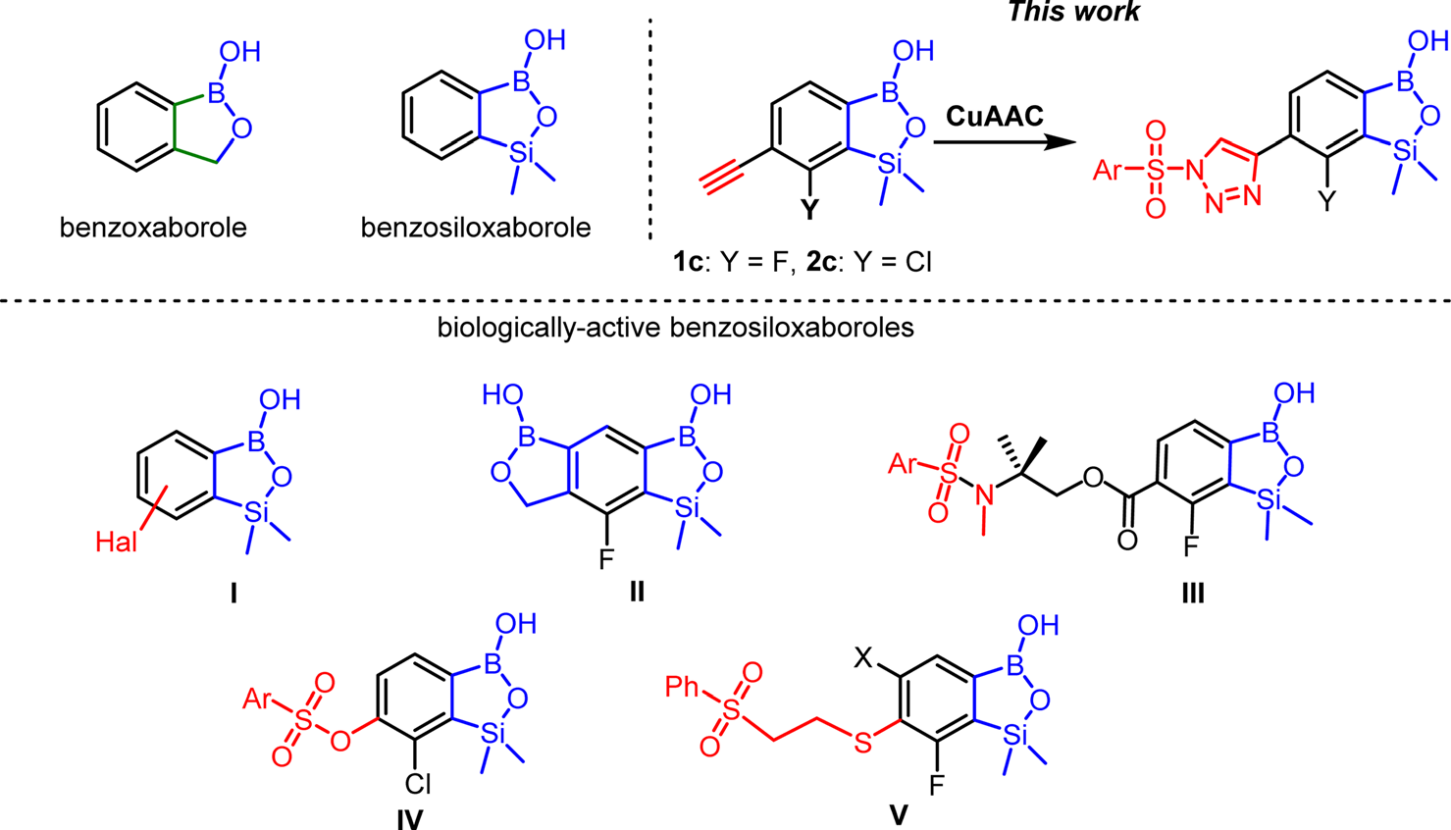
Examples of biologically active benzosiloxaboroles.

As a continuation of our studies on functionalized benzosiloxaboroles, we present herein the synthesis and in-depth structural characterization of ethynyl-substitited derivatives which highlights the effect of the π-hole on the boron atom on crystal packing. These relatively simple compounds were converted to derivatives bearing 1-arylsulfonyl-1,2,3-triazole-4-yl moiety. Such a structural design was inspired by our previous results showing that arylsulfonamido^17^ arylsulfonato,^18^ and (β-phenylsulfonyl)ethylthio^8^ substituted benzosiloxaboroles (Scheme 1, general structures III, IV, and V, respectively) exhibit high activity against the Gram-positive cocci such as methicillin-sensitive *Staphylococcus aureus* (MSSA), methicillin-resistant *S. aureus* (MRSA), *Enterococcus faecalis* and *Enterococcus faecium*. Based on the presented literature background,^10–16^ we assumed that the introduction of 1,2,3-triazole spacer between arylsulfonyl group and benzosiloxaborole might have further positive impact on the antimicrobial potency.

**Scheme 2:**
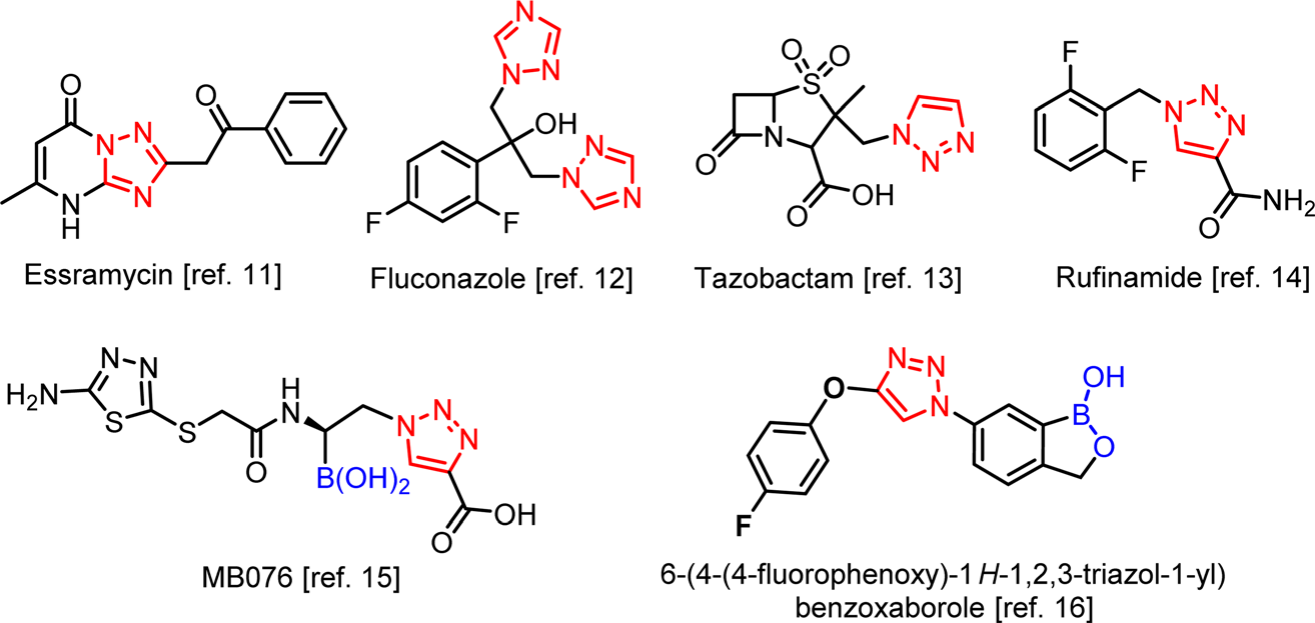
Biologically active compounds comprising 1,2,4- and 1,2,3-triazole rings including recent examples of organoboron compounds.

## 2 Results and discussion

### 2.1 Synthesis and crystal structure of 6-ethynyl-7-halogen substituted benzosiloxaboroles 1c and 2c

**Scheme 3:**
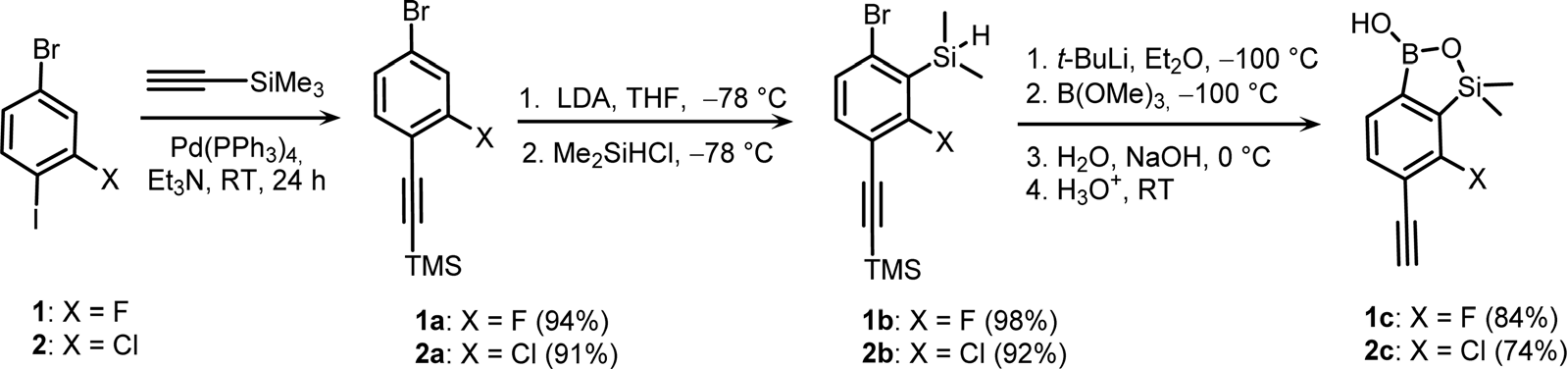
Synthesis of 6-ethynyl-7-halogen substituted benzosiloxaboroles 1c and 2c.

In a three-step procedure, 4-bromo-2-fluoro-1-iodobenzene 1 (Scheme 3) was converted to the respective TMS-protected ethynyl derivative 1a*via* Sonogashira coupling as reported previously^19^ followed by deprotonation with LDA at −78 °C and trapping of corresponding aryllithium intermediates with Me_2_Si(H)Cl to give arylsilane 1b in high yield. The lithiation occurred regioselectively at the 3-position flanked by two halogens which is activated by a strong cumulated *ortho*-acidifying effect of those two substituents.^20^ Compound 1b was subjected to Br/Li exchange with *t*-BuLi in Et_2_O at −100 °C followed by trapping with B(OMe)_3_. The hydrolysis effected with 1 M NaOH/H_2_O mixture resulted in a cleavage of the Si–H bond which occurs rapidly due to *ortho*-assistance of the anionic boronate group.^21^ The strongly alkaline conditions result also in removal of the trimethylsilyl group leading directly to the targeted derivative 1c which is the first example of the ethynyl-substituted benzosiloxaborole (Scheme 3). 7-Chloro-6-ethynyl derivative 2c was obtained using a similar three-step protocol starting with 4-bromo-2-chloro-1-iodobenzene 2. The overall yields of 1c and 2c were 77 and 62%, respectively. Both products were isolated as powders well soluble in most organic solvents. The ^1^H and ^13^C NMR spectra of 1c and 2c are in agreement with their structures whilst ^11^B NMR resonances at *ca.* 30 ppm are typical of other benzosiloxaboroles.^6^ It is worth noting that according to our best knowledge there are no published examples of ethynyl-substituted benzoxaboroles, *i.e.*, direct analogues of 1c and 2c.

### 2.2 Crystal structures 1c and 2c: the role of C(π)⋯B interactions

**Fig. 1:**
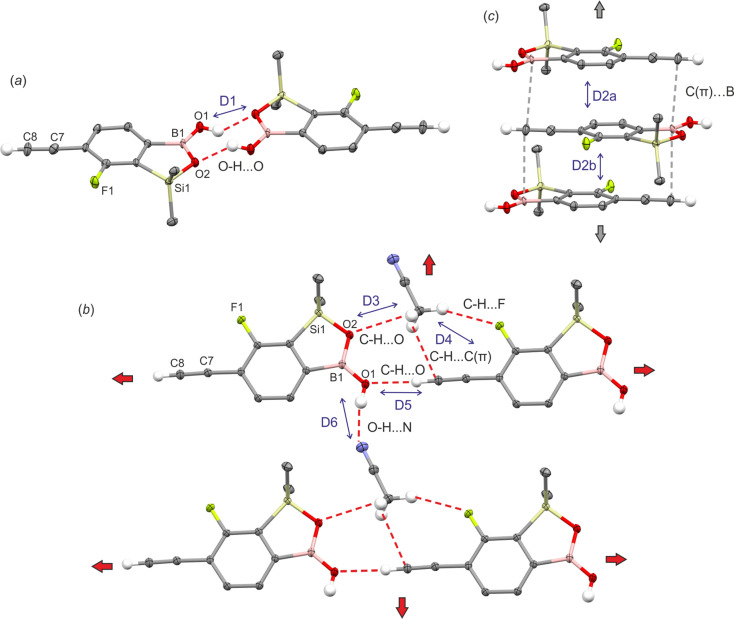
Fragments of crystal structures of 1c and 1c·MeCN showing the formation of (a) centrosymmetric hydrogen-bonded dimer in 1c, (b) molecular layer in 1c·MeCN and (c) columnar motifs (1c, 2c and 1c·MeCN) held by C(π)⋯B interactions. Note that two different D2 motifs (D2a and D2b) are distinguished in case of 1c and 2c. For 1c·MeCN the symmetry center appears between each pair of molecules within D2 motif.

**Fig. 2:**
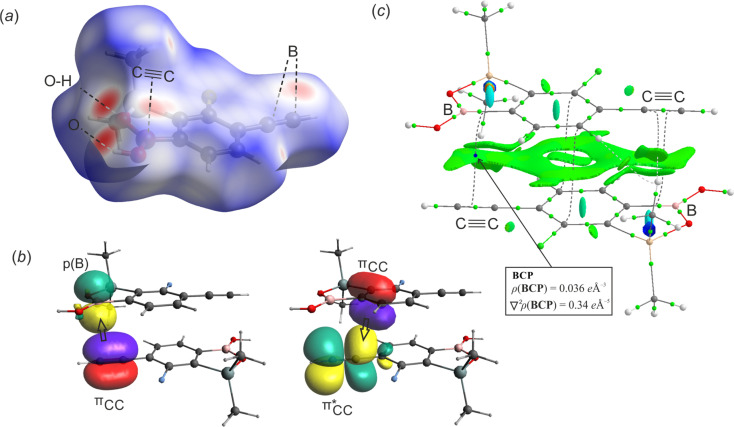
(a) Hirshfeld surfaces generated for 1c molecule with mapped *d*_norm_ property value over the range −0.50 to 1.30. (b) NBO orbitals participating in the intermolecular interaction with ethynyl group. (c) Combined QTAIM and NCI analysis of 1c. Bond critical points are represented by yellow spheres, BCP corresponding to C(π)⋯B interaction is represented by blue sphere. The color scale for the NCI plot is −0.01 < sign(*λ*_2_)*ρ* < 0.01; Reduced gradient density (RGD) isosurface of 0.3. The green color of RGD surface represents weak attractive interaction.

The geometry of benzosiloxaborole core in 1c is essentially the same as in related compounds reported by us previously.^9^ The supramolecular assembly of 1c shows centrosymmetric hydrogen-bonded (HB) dimers (O⋯O distance of 2.784(1) Å, motif D1, Fig. 1a) typical of boronic acid derivatives^22^ including most of benzoxaboroles and many benzosiloxaboroles.^6,22^ However, the molecules within a dimer are not coplanar but they are significantly displaced one to another so the angle between mean planes defined by the four O atoms of the dimeric motif and the atoms of the siloxaborole ring is 26.7°. Such geometrical deformations are also reflected in decreased interaction energy between molecules. According to DFT calculation performed at M06-2X/6-311++G(d,p) level of theory, the D1 dimer interaction energy in 1c is equal to 53.4 kJ mol^−1^, *i.e.*, lower compared to typical HB dimers in benzoxa- and benzosiloxaboroles (∼58 kJ mol^−1^). In addition, boron atoms show characteristic interactions with the π-density of two ethynyl groups of neighboured molecules (motifs D2a and D2b, Fig. 1c) characterized by respective intermolecular B⋯C(terminal) contacts of 3.394(2) (motif D2a) and 3.626(2) Å (motif D2b). The C(π)⋯B interactions, which can be considered as a type of π-hole triel bonds,^23^ are well visible on the Hirshfeld surfaces mapped over the *d*_norm_ property as shown in Fig. 2a. Specifically, the two major red spots are associated to O–H⋯O hydrogen bonds, while the two other red spots visible above the boron atom and ethynyl group correspond to C(π)⋯B interactions. Overall, the propagation of D2 dimeric motifs along the [100] direction leads to the formation of a 1D columnar assembly (Fig. 1c). The important contribution from π-stacking interaction between parallel aromatic rings should be also encountered thus giving rise to quite significant D2a and D2b dimer interaction energies of 34.5 and 37.3 kJ mol^−1^, respectively (Table 1).

**Table 1:** The main structural motifs and interaction energies in crystal structures of 1c, 1c·MeCN and 2c

Structure	Motif	Interaction	*d*_X⋯Y_ a/Å	*E*_int_ b/kJ mol^−1^
1c	D1	O–H⋯O	2.784(1)	53.4
	D2a	C(π)⋯B	3.394(2)	34.5
	D2b	C(π)⋯B	3.626(2)	37.3
2c	D1	O–H⋯O	2.793(2)	50.7
	D2a	C(π)⋯B	3.362(2)	41.4
	D2b	C(π)⋯B	3.640(2)	42.2
1c·MeCN	D2	C(π)⋯B	3.528(2)	45.3
	D3	C–H⋯O	3.189(2)	24.0
	D4	C–H⋯F	3.542(2)	8.3
		C–H⋯C(π)	3.267(2)	
	D5	C–H⋯O	3.155(1)	10.0
	D6	O–H⋯N	2.853(2)	28.9

a The intermolecular distance between heavy atoms.

b Interaction energies calculated at M06-2X/6-311++G(d,p) level of theory, positions of non-hydrogen atoms were retained from the crystal structure, and positions of hydrogen atoms were fully optimized.

In order to get deeper insight into the nature of C(π)⋯B interaction we have performed natural bond orbital (NBO) analysis and, in parallel, topological analysis of electron density within quantum theory of atoms in molecules (QTAIM) approach. The energetic contributions of intermolecular donor–acceptor interactions was estimated by 2nd-order perturbation theory (Table S1, ESI†). It comes out that the formation of C(π)⋯B interaction mostly originates from electron donation from ethynyl π_CC_ orbital to empty p_B_ orbital (orbital interaction energy *E* = 5.1 kJ mol^−1^ in D2a and *E* = 2.6 kJ mol^−1^ in D2b dimers, Fig. 2b). It is supported by the back-donation from phenyl π-electron density (represented by localized π_CC_ orbital) to antibonding π*_CC_ of ethynyl group (*E* ≈ 1.0 kJ mol^−1^). In addition, the non-covalent interaction index (NCI) was calculated for 2Da dimer. It revealed the typical pattern of π-stacking interactions reflected by the green area covering almost the whole interface between two adjacent aromatic rings (Fig. 2c). The reduced gradient density (RGD) isosurface is extended on the regions corresponding to the C(π)⋯B contact confirming the weak attractive character of this interaction. The QTAIM analysis revealed the appearance of bond critical point (BCP) located in-between the ethynyl group and boron atom with the electron density (*ρ*) of 0.036 e Å^−3^ and negative Laplacian (∇^2^*ρ*) of 0.34 e Å^−5^. The amount of electron density at BCP is slightly lower compared to the value found at BCP in model BCl_3_–ethynyl complex (*ρ* = 0.045 e Å^−3^) studied by Grabowski,^24^ and lies in the range of typical density values for two stacking aromatic rings (0.03–0.07 e Å^−3^).^25^ At this point, it should be noted that the bond path does not directly connect the ethynyl triple bond and boron atom but it ends at BCP of the B–C bond. Similar effect was already described for C=C(π)⋯B interaction in the crystal structure of (*E*)-(4-phenylbut-1-en-1-yl)boronic acid.^26^ The bond path might not be revealed from QTAIM analysis despite an attractive character of the C(π)⋯B interaction. Moreover, the presence of electron rich oxygen atoms at the boron atom may hinder the formation of the mentioned bond path.

The crystallization of 1c in acetonitrile afforded monosolvate 1c·MeCN. Unlike crystal structures of most benzoxaboroles including the case of non-solvated 1c, in 1c·MeCN the BOH group adopts anti conformation. The O atom of this group serves as a hydrogen bond acceptor for the ethynyl H atom of a neighbored molecule (*d*_O⋯H_ = 2.21 Å, *d*_C⋯O_ = 3.155(1) Å, motif D5) which results in the formation of a chain running along the *c* axis (Fig. 1b). The chains are cross-linked through interactions with MeCN molecules. First, the H atom of the BOH group is involved in O–H⋯N hydrogen bond (*d*_N⋯H_ = 2.03 Å, *d*_O⋯N_ = 2.853(2) Å, motif D6). In addition, the methyl group of the solvent guest molecule is anchored to two molecules of 1c through weaker C–H⋯F (*d*_C⋯F_ = 3.542(2) Å, motif D4), C–H⋯O (*d*_C⋯O_ = 3.189(2) Å, motif D3) and C–H⋯C(π-ethynyl) (*d*_C⋯C_ = 3.267(2) Å, as a part of motif D4) interactions. This results in a creation of a layer parallel to the (010) plane. The layers are generally assembled through π-stacking interactions but again characteristic intermolecular B⋯C(terminal) symmetrical contacts of 3.528(2) Å with the π-density of two ethynyl groups of neighboured molecules from both adjacent layers are observed. The interaction energy between molecules within D2 dimer is quite significant and equal to 45.3 kJ mol^−1^.

Compound 2c forms non-solvated crystals both from CHCl_3_ and MeCN solution. It is isostructural with 1c which means that the replacement of fluorine with chlorine has a marginal effect on the supramolecular assembly. As for 1c, centrosymmetric dimers are formed (O⋯O) distance of 2.793(2) Å, *i.e.*, by only 0.005 Å longer compared to (1c) and molecules within a dimer are significantly displaced one to another at the same angle of 25.9° as for 1c. Owing to the longer O⋯O distance, the interaction energy between molecules is slightly lower compared to 1c (50.7 kJ mol^−1^). The C(π)⋯B interactions are characterized by respective intermolecular B⋯C(terminal) contacts of 3.362(2) and 3.640(2) Å, *i.e.*, distance alternation is slightly smaller than for 1c. This resulted in increased intermolecular interaction energy for D2a (41.4 kJ mol^−1^) and D2b (42.2 kJ mol^−1^) dimers. Finally, C(π)⋯B orbital interaction energies and AIM parameters at corresponding BCPs are comparable for all studied systems (Tables S1.3 and S1.4, ESI†).

### 2.3 The use of 1c in Cu(i)-catalyzed 1,3-dipolar cycloaddition

**Scheme 4:**
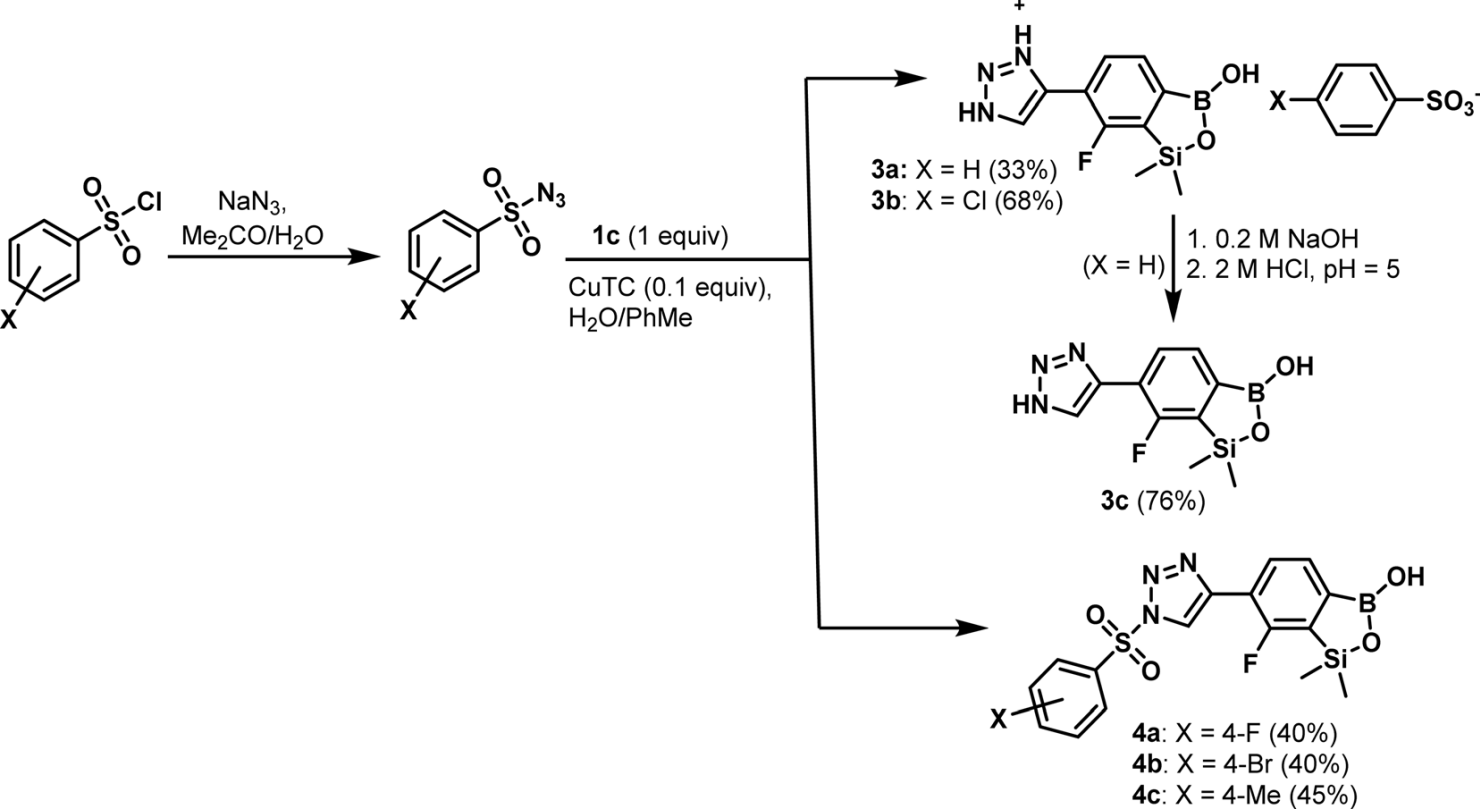
Application of Cu(i)-catalyzed azide–alkyne 1,3-dipolar cycloaddition for the synthesis of 1,2,3-triazole-4-yl functionalized benzosiloxaboroles 3a–3c and 4a–4c.

Compound 1c was utilized in Cu(i)-catalyzed azide–alkyne 1,3-dipolar cycloaddition (CuAAC) reactions with selected organic azides with a special emphasis on arylsulfonyl azides (easily accessible from the nucleophilic substitution of respective chlorides with NaN_3_ in acetone^27^). Previously, thermally induced azide–alkyne cycloaddition was reported for the preparation of a some boronated 1,2,3-triazoles.^28^ There are only a few reports on arylboronic-triazole conjugates up-to-date. For the first time, such compounds synthesized *via* CuAAC were reported by Hall *et al.*^29^ Kumar *et al.* synthesized a library of 1*H*-1,2,3-triazole-tethered 4-aminoquinoline-benzoxaborole hybrids and aryl-substituted benzoxaborole analogues. Obtained products were screened for their anti-plasmodial efficacy against both chloroquine-susceptibility 3D7 and chloroquine-resistant W2 strains of *P. falciparum*.^30^ The Passerini three component selective synthesis of benzoxaboroles with pendant tetrazole substituents shows also some resemblance to the present work due to the use of boronic acid and azide precursors.^31^ The standard CuSO_4_/sodium ascorbate catalytic system^32^ proved not effective in our CuAAC-based protocol utilizing 1c and various arylsulfonyl azides as starting materials. In contrast, Cu(i) thiophene-2-carboxylate (CuTC, 0.1 equiv.) proved more effective as a catalyst while the reactions were conducted in a biphasic water/toluene mixture (1 : 1).^33^ However, the use of simple benzenesulfonyl azide did not afford the expected cycloaddition product. Instead, the respective 4-(7-fluoro-3-hydroxy-1,1-dimethyl-1,3-dihydrobenzo[*c*][1,2,5]oxasilaborol-6-yl)-1*H*-1,2,3-triazol-3-ium salt with the benzenesulfonate counterion 3a, resulting from the hydrolytic abstraction of benzenesulfonyl group, was isolated. An analogous result was obtained with 4-chlorophenylsulfonyl azide. Treatment of 3a with aq. NaOH followed by careful acidification with aq. HCl to pH = 5 afforded the neutral product 3c. However, in selected cases it was possible to isolate intermediates, *i.e.*, the elaborated protocol allowed for the synthesis of a series of functionalized benzosiloxaboroles 4a–4c (Scheme 4). The products of studied CuAAC reactions were obtained in moderate yields (33–68%). Overall, the cycloaddition reaction proceeds smoothly but it is difficult to control the subsequent potential abstraction of arylsulfonyl moiety.

**Fig. 3:**
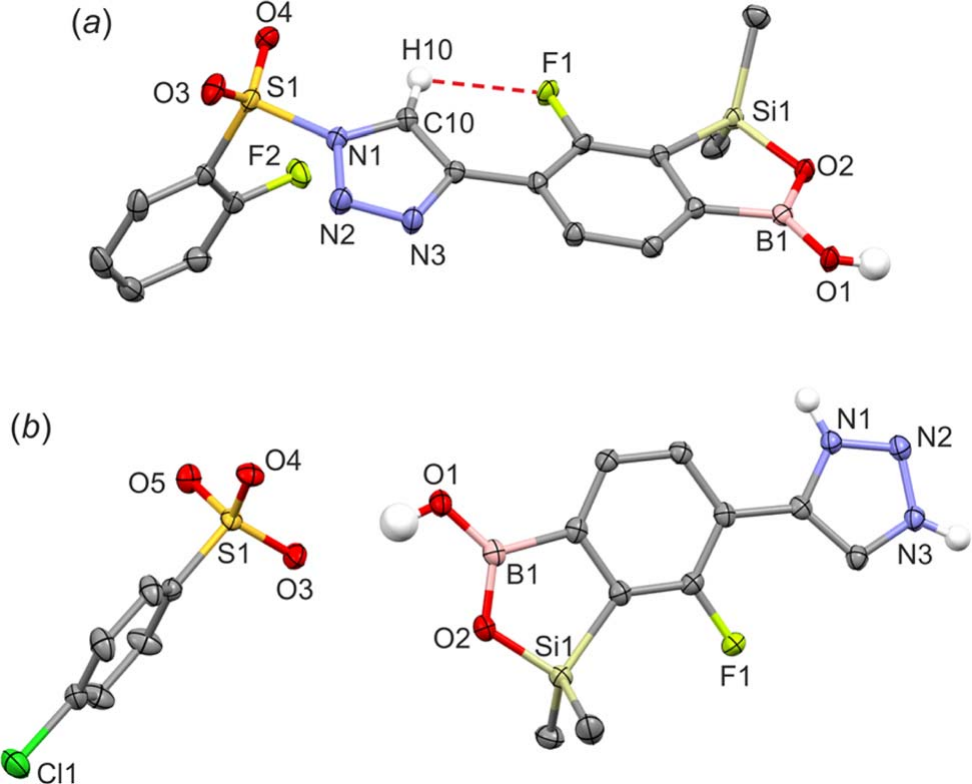
The molecular structures of (a) 4d and (b) 3b.

**Fig. 4:**
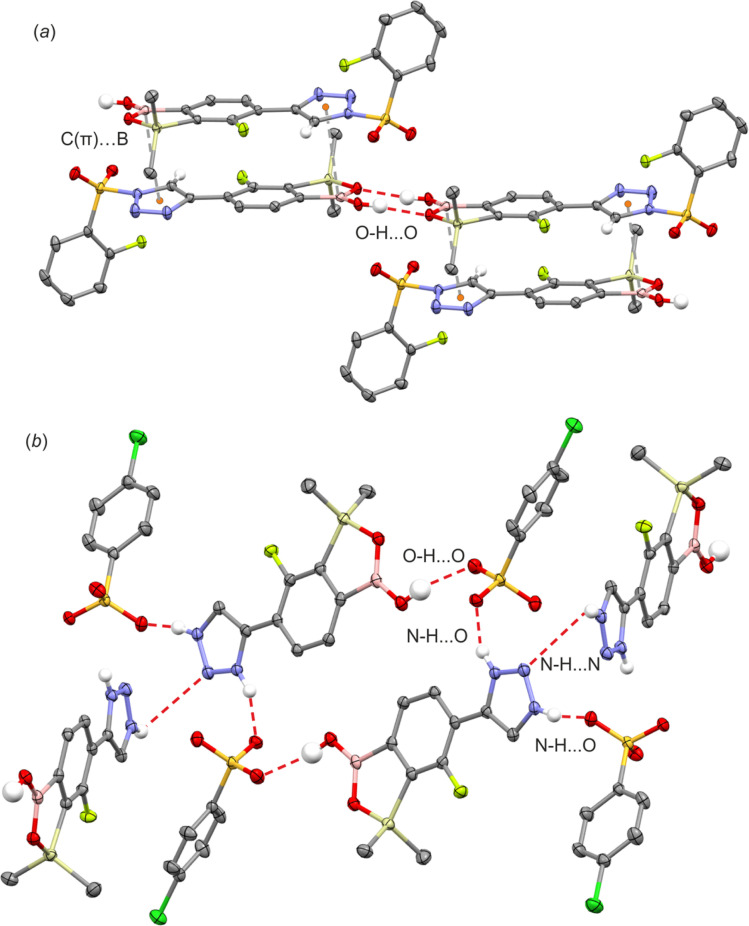
Fragments of the supramolecular structures of (a) 4d and (b) 3b.

All products were characterized by ^1^H, ^13^C and ^19^F NMR spectroscopy as well as HRMS. In the NMR spectra of 4a–4c the signal of the proton at the 5-position of 1,2,3-triazole ring at *ca.* 8.8–9.1 ppm appears always as a doublet with a coupling constant of *ca.* 3.5 Hz which may be attributed the existence of a through-space ^1^H–^19^F coupling with the fluorine atom. In addition, compound 4d bearing the pendant 2-fluorophenylsulfonyl moiety was characterized by single crystal X-diffraction (Fig. 3a) as a monosolvate 4d CHCl_3_. It should be noted that we were unable to obtain pure bulk 4d and the crystal was grown from the mixture containing mainly a respective ionic species analogous to 3a–3b. In the molecule 4d the triazole ring is essentially coplanar with the benzosiloxaborole core (the interplanar angle of benzene and triazole ring is 10.5°) as that conformation apparently gains stabilization owing to the short intramolecular C10(H)⋯F1 contact of 2.37 Å which is in agreement with ^1^H NMR data. Compound 4d also forms dimers but the angle of displacement of molecules within a dimer (defined above for 1c) is only 10.3° (Fig. 4a). The packing also shows stacking of the adjacent molecules characterized by interactions of the boron atom with the π-density of the triazole rings. The distance between the B atom and triazole ring centroid is 3.61 Å. The partially disordered CHCl_3_ molecules are closely paired^34^ in the crystal structure cavities and show H-bonding interactions with triazole rings as the C19(H)⋯N3 distance is relatively short (3.193 Å).

The geometry of the cationic part of compound 3b is similar to that of the analogous fragment in 4d (Fig. 3b). Unlike 4d, the cations do not assemble through the formation of centrosymmetric dimeric motifs involving B(O)OH groups. Instead, two types of charge-assisted NH⋯O hydrogen bonds (*d*_N⋯O_ = 2.684 Å, *d*_N⋯O_ = 2.669 Å) link cationic triazolium moieties with two neighbored arylsulfonate anions (Fig. 4b). This is complemented by another hydrogen bond formed between the BOH group and the third oxygen atom of the sulfonate group (*d*_O⋯O_ = 2.882 Å). Overall, this results in a layer network located symmetrically with respect to the (100) plane. The layers interact by means of weak C–H⋯Cl interactions of methyl groups with chloro substituents. In addition, we attempted to grow single crystals of the neutral compound 3c. However, we obtained invariably an amorphous material which can be attributed to dynamic equilibria between various forms of 3c, presumably involving proton exchange at the triazole ring and/or aggregation processes involving formation of N–B coordination bonds. This is indicated by strongly broadened signals in the ^1^H NMR spectrum of 3c (Fig. S3.17†).

**Scheme 5:**
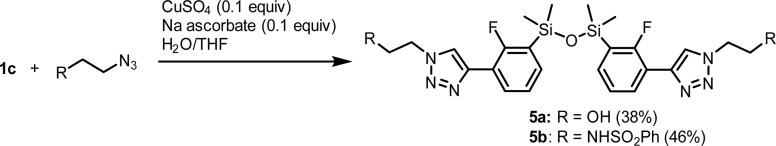
Formation of functionalized 1,3-diaryl-1,1,3,3-tetramethyldisiloxanes 5a–5b.

**Fig. 5:**
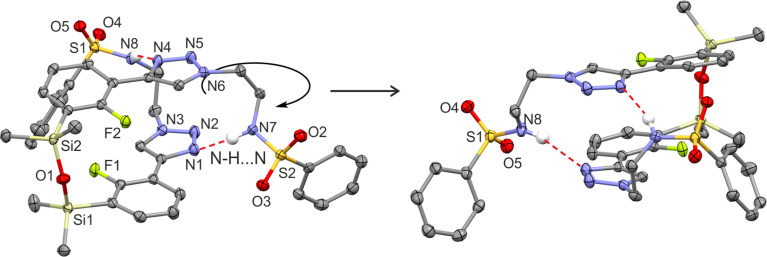
The molecular structure of 5b viewed from two different perspectives.

We have also studied the reactions of 1c with functionalized alkyl azides XCH_2_CH_2_N_3_ (X = OH, NHSO_2_Ph). Since CuTC was not effective as a catalyst in both cases, we decided to use the classical system CuSO_4_/sodium ascorbate in H_2_O/THF mixture. This approach did not result in expected benzosiloxaboroles as cycloaddition reactions were accompanied by extensive protodeboronation, presumably catalyzed by Cu^2+^ cations. Thus, the respective structurally extended 1,3-diaryl-1,1,3,3-tetramethyldisiloxanes 5a–5b were isolated in reasonable yields as isolable products of a dehydrative condensation of initially formed aryldimethylsilanols (Scheme 5). This was confirmed for 5b by X-ray crystallography showing a specific curled conformation of the molecule stabilized through two intramolecular NH⋯N hydrogen bonds (*d*_N⋯N_ = 2.922 Å, *d*_N⋯N_ = 2.929 Å) linking sulfonamide groups and triazole rings (Fig. 5). In addition, π–π stacking interactions of internal benzene and triazole rings play also some role (in the former case, it would be probably more precise to invoke mutual C–F dipole–dipole or C–F⋯π interactions).

### 2.4 Antimicrobial activity

Taking into account the direct antimicrobial activity of various benzosiloxaborole derivatives demonstrated previously^6,7,17,18,35^ in the current research we determined the activity of the newly synthesized compounds against a wide panel of Gram-positive, Gram-negative bacteria and yeasts strains. All obtained results were summarized in Tables S2.1 and S2.3 in the ESI.† 8 of 10 tested compounds displayed moderate to weak activity against Gram-positive cocci, with MICs ranging from 12.5 to 200 mg L^−1^ (Tables 2 and S2.1†). Among these active compounds, derivatives 2c and 3c were highly active against standard staphylococci, including MRSA, with MIC 12.5 mg L^−1^. Despite the approval of new drugs and actions taken around the world to limit the spread of drug-resistant strains, MRSA strains are still one of the most common pathogens causing nosocomial infections. MRSA strains have been entered on the WHO list of the most dangerous pathogens for humans.^36^ It is worth emphasizing that MRSA strains are resistant to almost all β-lactams (except ceftaroline and ceftobiprole) and are often resistant to many other groups of antibiotics as macrolides, tetracyclines, aminoglycosides, and fluoroquinolones.^37,38^ However, the most promising compounds 2c and 3c, showed from 6- to 12-fold less activity than linezolid as a reference agent. Linezolid is a representative of a relatively new group of drugs active against cocci, including MRSA strains.^38^ In our previous publication, we reported that among benzenesulfonato benzosiloxaborole derivatives display the highest potency (MICs 0.39–3.12 mg L^−1^) against *Staphylococcus* spp.^18^ However, expanding the structure of compounds tested in this study with arylsulfonyl group resulted in a reduction in activity against staphylococci and enterococci (from 2- to 4-fold reduction in the MIC values of compounds 3a, 3b, 4b, and 4c, compared to 3c) (Table 2). It seems that this can be attributed to the facile abstraction of the arylsufonyl group under aqueous conditions resulting in the formation of respective 1,2,3-triazolium arylsulfonates where only the cationic part comprising the siloxaborole ring exhibits antibacterial acitivity. For most compounds, the minimal bactericidal concentration (MBC) values were high (>400 mg L^−1^). Notably, the so-called Eagle effect (also known as the paradoxical growth reported for various antibiotics)^39^ was observed in the case of *S. aureus* ATCC 6538P (for 1c, 2c and 3c) and *S. epidermidis* ATCC 12228 (for 1c and 3c). It implies occurrence of two MBC values as determined by us previously for other substitued benzosiloxaboroles.^17,18^ The first MBC values were 2-fold higher than the MIC values. Furthermore, a progressive increase in the number of surviving bacteria was observed at higher concentrations followed by a subsequent decrease. If the bacterial population was reduced again to the MBC threshold, a second MBC value (at 200 ≥ 400 mg L^−1^) could be determined.

**Table 2:** The MIC and MBC values of selected new compounds against standard Gram-positive strains^a^

Agent tested	MIC^b^ in mg L^−1^ [MBC in mg L^−1^]				
	*S. aureus* ATCC 6538P	*S. aureus* ATCC 43300 MRSA	*S. epidermidis* ATCC 12228	*E. faecalis* ATCC 29212	*E. faecium* ATCC 6057
1c/147	25 [*50*/*400*]^c^	25 [50]	**12.5** [*25*/>*400*]^c^	100	100
2c/253	**12.5** [*25*/*200*]^c^	**12.5** [25]	**12.5** [400]	50	50
3a/166	25	50	50	200	200
3b/170	50	50	50	200	200
3c/254	**12.5** [*25*/>*400*]^c^	**12.5** [25]	**12.5** [*25*/>*400*]^c^	100	50
4a/167	25	50	50	200	200
4b/169	25	50	50	200	200
4c/171	25	50	50	200	200
LIN^d^	1 [>128]	2 [>128]	1 [>128]	2 [>128]	2 [>128]

a The highest activity against Gram-positive bacteria indicated by the low MIC values (≤12.5 mg L^−1^) is shown in boldface. (—) the inhibition zone was not observed in the disc-diffusion method. The diameter of the paper discs was 9 mm.

b Only the MBC values ≤400 mg L^−1^ are presented.

c The Eagle effect^39^ was observed during the determination of the MBC value of the same tested agents against *Staphylococcus* spp. strains. The Eagle effect is shown in the italic face.

d LIN, linezolid was used as a reference agent active against Gram-positive bacteria. The diameter of a commercial disc containing 0.03 mg of linezolid was 6 mm; the MIC of linezolid was determined according to the CLSI recommendations.^40^

Only 1c and 2c displayed weak activity against Gram-negative rods with MICs ranging from 200 to >400 mg L^−1^ (Table S2.2†). Considering that resistance of Gram-negative rods is frequently associated with efflux pump activity,^41^ we also determined the MICs of newly compounds in the presence of efflux pump inhibitor, *i.e.*, phenylalanine-arginine-β-naphthylamide (PAβN).^42^ In the case of *Escherichia coli*, *Klebsiella pneumoniae*, *Serratia marcescens* and *Stenotrophomonas maltophilis* strains the MICs of compounds 1c, 2c and 3c are reduced at least 4-fold in the presence of PAβN. This means that these compounds are actively removed by efflux pumps of bacterial cells.

Considering previously reported antifungal activity of some benzosiloxaboroles (with MICs ranging from 0.78–12.5 mg L^−1^)^6,7^ we investigated the activity of new derivatives against 7 standard yeast strains. The moderate antifungal activity was observed only for 1c and 2c with MICs ranging from 12.5 to 400 mg L^−1^ (Table S2.3†).

## 3 Conclusions

In conclusion, a convenient protocol for the synthesis of halogenated benzosiloxaboroles 1c and 2c comprising reactive ethynyl substituents was elaborated. It should be noted that analogous functionalized benzoxaboroles are still unknown which strengthens the importance of presented results. Intriguingly, C(π)⋯B interactions seem to play a decivise in role in supramolecular organization of these compounds which is confirmed by the fact that it is retained in the crystal structure of the solvate 1c MeCN. This observation indicates a high potential of the ethynyl group in crystal engineering of boracyclic compounds. CuAAC reaction of 1c using arylsulfonyl azides gave rise to respective products 4a–4c bearing 1-(arylsulfonyl)-1*H*-1,2,3-triazol-4-yl functionalities. However, compounds of this type are rather prone to hydrolysis as evidenced by the isolation of 1,2,3-triazolium arylsulfonate salts 3a–3b and the neutral benzosiloxaborole 3c with attached 1,2,3-triazol-4-yl moiety. In turn, the analogous cycloaddition reactions with functionalized alkyl azides XCH_2_CH_2_N_3_ (X = OH, NHSO_2_Ph) were accompanied by extensive protodeboronation and thus furnished respective 1,3-diaryl-1,1,3,3-tetramethyldisiloxanes 5a–5b as isolable products. Structural studies of final products were complemented by evaluation of antimicrobial actitivity which revealed that the ethynyl-substituted derivatives 1c and 2c are more potent antibacterial agents than structurally expanded 1,2,3-triazolyl derivatives. Based on previous findings, we assumed initially that the presence of arylsulfonyl end groups could be beneficial but it was definitely not the case. This might be rationalized by the tendency of these compounds to hydrolysis which can occur under physiological conditions. Further studies on the use of ethynyl-substituted benzosiloxaboroles as a platform for the construction of structurally extended systems including nucleoside conjugates are currently in progress and the results will be reported in due course.

## 4 Experimental section

### 4.1 General comments

Solvents used for reactions were dried by heating to reflux with sodium/benzophenone and distilled under argon. Starting materials including halogenated benzenes, ethynyltrimethylsilane, Pd(PPh_3_)_4_, CuI, chlorodimethylsilylane, alkyllithiums, diisopropylamine, trimethyl borate, sodium azide, CuTC, arylsulfonyl chlorides, as well as other reagents were used as received without further purification. In the ^13^C NMR spectra, the resonances of boron-bound carbon atoms were not observed in some cases as a result of their broadening by a quadrupolar boron nucleus. ^1^H and ^13^C NMR chemical shifts are given relative to TMS using residual solvent resonances. ^11^B and ^19^F NMR chemical shifts are given relative to BF_3_·Et_2_O and CFCl_3_, respectively.

### 4.2 Synthesis

#### 4.2.1 ((4-Bromo-2-fluorophenyl)ethynyl)trimethylsilane (1a)

A mixture of 4-bromo-2-fluoro-1-iodobenzene (39.1 g, 0.13 mol), ethynyltrimethylsilane (14.1 g, 0.14 mol), tetrakis(triphenylphosphine) palladium(0) (6.01 g, 5.20 mmol) and CuI (7.43 g, 39.00 mmol) in Et_3_N (180 mL) was stirred in under argon at room temperature for 24 h. It was then cooled to room temperature, and the saturated NH_4_Cl aqueous solution (500 mL) and EtOAc (200 mL) were added. After separation of the organic phase, the aqueous phase was extracted with ethyl acetate (2 × 150 mL), and the combined organic extracts are dried with anhydrous MgSO_4_ and concentrated on a rotary evaporator. The residue was subjected to column chromatography on silica with hexane as the eluent to give the product as a colorless oil (33.3 g, 94%). ^1^H NMR (400 MHz, CDCl_3_) *δ* 7.33–7.28 (m, 1H), 7.27–7.20 (m, 2H), 0.26 (s, 9H) ppm. ^13^C NMR (101 MHz, CDCl_3_) *δ* 162.6 (d, *J* = 256.6 Hz), 134.6 (d, *J* = 1.9 Hz), 127.3 (d, *J* = 3.8 Hz), 122.8 (d, *J* = 8.9 Hz), 119.2 (d, *J* = 24.3 Hz), 111.0 (d, *J* = 16.0 Hz), 101.5 (d, *J* = 3.4 Hz), 96.8, −0.2 ppm. ^19^F NMR (376 MHz, CDCl_3_) *δ* −107.03 (t, *J* = 8.0 Hz) ppm.

#### 4.2.2 ((4-Bromo-3-(dimethylsilyl)-2-fluorophenyl)ethynyl)trimethylsilane (1b)

A solution of 1a (15.3 g, 56.5 mmol) in THF (20 mL) was added dropwise at −75 °C for 15 min to a stirred solution of LDA, freshly prepared from diisopropylamine (9.0 mL, 62.1 mmol, 1.1 equiv.) and *n*-BuLi (2.5 M, 24.8 mL, 62.1 mmol, 1.1 equiv.) in THF (100 mL). After *ca.* 1 h of stirring at 75 °C, chlorodimethylsilane (7.5 mL, 67.7 mmol, 1.2 equiv.) was added slowly to a resulting yellow solution. The mixture was stirred at −75 °C for 15 min, and then it was allowed to warm to room temperature. The obtained white suspension was evaporated to dryness under reduced pressure. The residue was triturated with heptane (50 mL) followed by filtration. The yellow filtrate was evaporated under reduced pressure, and the residue was distilled under reduced pressure. The product 1b was obtained as a yellowish oil, bp 90–95 °C (5·10^−3^ mbar). Yield 18.1 g (98%). ^1^H NMR (400 MHz, CDCl_3_) *δ* 7.29 (d, *J* = 0.7 Hz, 1H), 7.28 (d, *J* = 1.4 Hz, 1H), 4.75 (dhept, *J* = 4.9, 3.9 Hz, 1H), 0.45 (dd, *J* = 3.9, 1.9 Hz, 6H), 0.26 (s, 9H) ppm. ^13^C NMR (101 MHz, CDCl_3_) *δ* 166.8 (d, *J* = 250.7 Hz), 135.5 (d, *J* = 2.3 Hz), 130.4 (d, *J* = 11.8 Hz), 128.7 (d, *J* = 3.7 Hz), 126.1 (d, *J* = 32.5 Hz), 110.6 (d, *J* = 21.1 Hz), 101.1 (d, *J* = 3.6 Hz), 97.3, −0.2, −3.3 (d, *J* = 4.3 Hz) ppm. ^19^F NMR (376 MHz, CDCl_3_) *δ* −91.48 ÷ −91.55 (m) ppm.

#### 4.2.3 6-Ethynyl-7-fluoro-1,1-dimethylbenzo[*c*][1,2,5]oxasilaborol-3(1*H*)-ol (1c)

A solution of *t*-BuLi (1.7 M in pentane, 55.5 mL, 0.094 mol, 1.5 equiv.) was added dropwise at −78 °C to Et_2_O (60 mL) under argon atmosphere. The solution was cooled to −100 °C, followed by a dropwise addition of a solution of 1b (20.7 g, 0.063 mol, 1.0 equiv.) in Et_2_O (60 mL) for 30 min. After *ca.* 30 min stirring at −100 °C, a thick suspension was formed. B(OMe)_3_ (14.0 mL, 0.126 mol, 2.0 equiv.) was added dropwise for 30 min and the mixture was warmed to −10 °C, quenched with 1 M NaOH/H_2_O (150 mL) and stirred at room temperature until the evolution of H_2_ ceased. The obtained white suspension was concentrated under reduced pressure in order to remove organic solvents and washed with heptane (150 mL). 1.5 M aq. H_2_SO_4_ was dropped to reach the pH 2–3 (100 mL), and a white solid was precipitated. It was filtered and washed with water (2 × 25 mL), heptane (2 × 25 mL), and dried *in vacuo* to give 1c as a white powder, mp: 110–113 °C. Yield 7.50 g (84%). ^1^H NMR (400 MHz, CDCl_3_) *δ* 7.59 (dd, *J* = 7.4, 6.4 Hz, 1H), 7.54 (dd, *J* = 7.3, 1.8 Hz, 1H), 5.25 (s, 1H), 3.36 (d, *J* = 0.7 Hz, 1H), 0.50 (s, 6H) ppm. ^13^C NMR (101 MHz, CDCl_3_) *δ* 164.7 (d, *J* = 251.4 Hz), 143.7, 136.5, 134.9 (d, *J* = 30.9 Hz), 127.2 (d, *J* = 3.4 Hz), 112.4 (d, *J* = 18.7 Hz), 83.3 (d, *J* = 3.1 Hz), 77.3, −0.8 ppm. ^11^B NMR (96 MHz, CDCl_3_) *δ* 29.6 ppm. ^19^F NMR (376 MHz, CDCl_3_) *δ* −101.42 (dd, *J* = 6.6, 2.2 Hz) ppm. HRMS (ESI, negative ion mode): calcd for C_10_H_9_BFO_2_Si^−^ [M − H]^−^ 219.0454; found 219.0450.

#### 4.2.4 ((4-Bromo-2-chlorophenyl)ethynyl)trimethylsilane (2a)

The synthesis was performed as described for 1a using 4-bromo-2-chloro-1-iodobenzene (12.7 g, 0.04 mol) as the starting material. The product was obtained as a colorless oil. Yield (10.5 g, 91%). ^1^H NMR (400 MHz, CDCl_3_) *δ* 7.55 (dd, *J* = 1.6, 0.7 Hz, 1H), 7.33 (dd, *J* = 8.3, 0.7 Hz, 1H), 7.31 (dd, *J* = 8.3, 1.6 Hz, 1H), 0.28 (s, 9H) ppm. ^13^C NMR (101 MHz, CDCl_3_) *δ* 137.2, 134.4, 132.1, 129.8, 122.7, 122.3, 101.7, 100.4, −0.1 ppm.

#### 4.2.5 ((4-Bromo-3-(dimethylsilyl)-2-chlorophenyl)ethynyl)trimethylsilane (2b)

The synthesis was performed as described for 1b using 2a (10.0 g, 0.035 mol) as the starting material. The product was obtained as a colorless oil. Yield (11.1 g, 92%). ^1^H NMR (400 MHz, CDCl_3_) *δ* 7.40 (d, *J* = 8.3 Hz, 1H), 7.30 (d, *J* = 8.3 Hz, 1H), 5.07 (hept, *J* = 3.9 Hz, 1H), 0.50 (d, *J* = 4.1 Hz, 6H), 0.28 (s, 9H) ppm. ^13^C NMR (101 MHz, CDCl_3_) *δ* 143.3, 138.6, 135.0, 131.6, 131.0, 123.0, 101.6, 101.2, 0.0, −2.5 ppm.

#### 4.2.6 6-Ethynyl-7-chloro-1,1-dimethylbenzo[*c*][1,2,5]oxasilaborol-3(1*H*)-ol (2c)

The synthesis was performed as described for 1c using 2b (10.3 g, 0.030 mol) as the starting material. The product was obtained as a beige powder. Yield (5.2 g, 74%). ^1^H NMR (400 MHz, CDCl_3_) *δ* 7.64 (d, *J* = 7.4 Hz, 1H), 7.61 (d, *J* = 7.5 Hz, 1H), 5.40 (broad s, 1H), 3.45 (s, 1H), 0.52 (s, 6H) ppm. ^13^C NMR (101 MHz, CDCl_3_) *δ* 150.0, 142.7, 139.2, 136.1, 129.5, 124.1, 83.9, 80.5 ppm. ^11^B NMR (96 MHz, CDCl_3_) *δ* 29.6 ppm. HRMS (ESI, negative ion mode): calcd for C_10_H_9_BClO_2_Si^−^ [M − H]^−^ 235.0159; found 235.0158.

#### 4.2.7 4-(7-Fluoro-3-hydroxy-1,1-dimethyl-1,3-dihydrobenzo[*c*][1,2,5]oxasilaborol-6-yl)-1*H*-1,2,3-triazol-3-ium benzenesulfonate (3a)

A glass vial was charged with copper(i) thiophene-2-carboxylate (CuTC, 0.15 mmol, 0.1 equiv. with respect to alkyne), water (5 mL), toluene (5 mL) and 1c (1.5 mmol, 1 equiv.). The reaction mixture was cooled in an ice-water bath. Subsequently, the benzenesulfonyl azide (1.5 mmol, 1 equiv.) was added slowly. The reaction mixture was allowed to warm to room temperature and stirred for 6 h. The reaction was diluted with saturated aq. NH_4_Cl (5 mL) and extracted into AcOEt (2 × 5 mL). The combined organics were dried (MgSO_4_) and filtered. The eluent was concentrated *in vacuo*. To remove remaining copper salts, the residue was redissolved in AcOEt and Cuprisorb® resin (1.0 g) was added. The mixture was stirred, filtered and concentrated *in vacuo*. The resulting solid was crystallized in a cold CHCl_3_/hexane mixture (1 : 3) and collected by filtration to afford the product. It was obtained as a white powder, mp: 183–186 °C (decomposition). Yield 0.20 g (33%). ^1^H NMR (400 MHz, DMSO-*d*_6_) *δ* 8.25 (d, *J* = 3.8 Hz, 1H), 8.17 (t, *J* = 7.4 Hz, 1H), 7.75 (dd, *J* = 7.4, 2.0 Hz, 1H), 7.61–7.58 (m, 2H), 7.33–7.30 (m, 3H), 0.45 (s, 6H) ppm. ^13^C NMR (101 MHz, DMSO-*d*_6_) *δ* 160.4 (d, *J* = 246.7 Hz), 148.0, 144.2 (broad), 139.5, 135.5 (d, *J* = 31.9 Hz), 130.5 (*J* = 2.1 Hz), 128.6, 128.3 (*J* = 3.0 Hz), 127.8, 125.5, 119.5 (d, *J* = 16.0 Hz), −0.5 ppm. ^11^B NMR (96 MHz, DMSO-*d*_6_) *δ* 29.9 ppm. ^19^F NMR (376 MHz, DMSO-*d*_6_) *δ* −105.71 ppm. HRMS (ESI, positive ion mode): calcd for C_10_H_12_BFN_3_O_2_Si^+^ [M]^+^ 264.0770; found 264.0771.

#### 4.2.8 4-(7-Fluoro-3-hydroxy-1,1-dimethyl-1,3-dihydrobenzo[*c*][1,2,5]oxasilaborol-6-yl)-1*H*-1,2,3-triazol-3-ium 4-chlorophenylsulfonate (3b)

White powder, mp: 202–204 °C (dec.). Yield 0.45 g (68%). ^1^H NMR (400 MHz, DMSO-*d*_6_) *δ* 8.25 (d, *J* = 3.8 Hz, 1H), 8.17 (t, *J* = 7.3 Hz, 1H), 7.75 (dd, *J* = 7.5, 1.9 Hz, 1H), 7.64–7.60 (m, 2H), 7.39–7.35 (m, 2H), 0.45 (s, 6H) ppm. ^13^C NMR (101 MHz, DMSO-*d*_6_) *δ* 160.4 (d, *J* = 246.6 Hz), 147.1, 144.2 (broad), 139.5, 135.5 (d, *J* = 31.8 Hz), 133.1, 130.5 (d, *J* = 2.1 Hz), 128.6, 128.3 (d, *J* = 3.0 Hz), 127.8, 127.5, 119.5 (d, *J* = 16.0 Hz), −0.5 ppm. ^11^B NMR (96 MHz, DMSO-*d*_6_) *δ* 29.9 ppm. ^19^F NMR (376 MHz, DMSO-*d*_6_) *δ* −105.98 ppm. HRMS (ESI, positive ion mode): calcd for C_10_H_12_BFN_3_O_2_Si^+^ [M]^+^ 264.0770; found 264.0771.

#### 4.2.9 4-(7-Fluoro-3-hydroxy-1,1-dimethyl-1,3-dihydrobenzo[*c*][1,2,5]oxasilaborol-6-yl)-1*H*-1,2,3-triazole (3c)

Compound 3a (0.39 g, 1.0 mmol) was dissolved in aq. NaOH (0.2 M, 7 mL). The solution was filtered and 2 M aq. HCl was added dropwise. The precipitated solid was filtered, washed with water and dried to give the product as a beige powder. Yield 0.20 g (76%). ^1^H NMR (400 MHz, DMSO-*d*_6_) *δ* 9.36 (s, 1H), 8.35–8.05 (broad, 2H), 7.74 (d, *J* = 7.3 Hz, 1H), 0.45 (s, 6H) ppm. ^11^B NMR (96 MHz, DMSO-*d*_6_) *δ* 19.9 ppm. HRMS (ESI, positive ion mode): calcd for C_10_H_12_BFN_3_O_2_Si^+^ [M]^+^ 264.0770; found 264.0771.

#### 4.2.10 7-Fluoro-1,1-dimethyl-6-(1-(4-fluorophenylsulfonyl)-1*H*-1,2,3-triazol-4-yl)benzo[*c*][1,2,5]oxasilaborol-3(1*H*)-ol (4a)

White powder, mp: 133–137 °C (dec.). Yield 0.25 g (40%). ^1^H NMR (400 MHz, acetone-*d*_6_) *δ* 8.83 (d, *J* = 3.3 Hz, 1H), 8.41–8.36 (m, 2H), 8.31 (dd, *J* = 7.6, 6.8 Hz, 1H), 8.30 (s, 1H), 7.76 (dd, *J* = 7.5, 1.9 Hz, 1H), 7.58–7.53 (m, 2H), 0.48 (s, 6H) ppm. ^13^C NMR (101 MHz, acetone-*d*_6_) *δ* 167.9 (d, *J* = 257.8 Hz), 161.7 (d, *J* = 247.5 Hz), 145.7, 141.9, 133.1 (d, *J* = 10.4 Hz), 131.3 (d, *J* = 1.7 Hz), 128.9 (d, *J* = 3.3 Hz), 123.9 (d, *J* = 12.8 Hz), 119.2 (d, *J* = 16.0 Hz), 119.0 (d, *J* = 12.1 Hz), 118.5 (d, *J* = 23.6 Hz), −0.6 ppm. ^11^B NMR (96 MHz, acetone-*d*_6_) *δ* 29.8 ppm. ^19^F NMR (376 MHz, acetone-*d*_6_) *δ* −101.32 ÷ −101.39 (m), −105.56 ÷ −105.60 (m) ppm. HRMS (ESI, positive ion mode): calcd for C_16_H_15_BF_2_N_3_O_4_SSi^+^ [M + H]^+^ 422.0608; found 422.0608.

#### 4.2.11 7-Fluoro-1,1-dimethyl-6-(1-(4-bromophenylsulfonyl)-1*H*-1,2,3-triazol-4-yl)benzo[*c*][1,2,5]oxasilaborol-3(1*H*)-ol (4b)

White powder, mp: 133–136 °C (dec.). Yield 0.29 g (40%). ^1^H NMR (400 MHz, acetone-*d*_6_) *δ* 8.84 (d, *J* = 3.3 Hz, 1H), 8.31 (dd, *J* = 7.6, 7.0 Hz, 1H), 8.22–8.18 (m, 2H), 8.00–7.96 (m, 2H), 7.76 (dd, *J* = 7.5, 1.9 Hz, 1H), 0.48 (s, 6H) ppm. ^13^C NMR (101 MHz, acetone-*d*_6_) *δ* 161.8 (d, *J* = 247.5 Hz), 142.0 (d, *J* = 3.5 Hz), 136.8 (d, *J* = 31.9 Hz), 136.2, 134.4, 132.0, 131.3 (d, *J* = 1.6 Hz), 131.2, 128.9 (d, *J* = 3.1 Hz), 124.0 (d, *J* = 12.9 Hz), 119.1 (d, *J* = 16.1 Hz), −0.6 ppm. ^11^B NMR (96 MHz, acetone-*d*_6_) *δ* 29.8 ppm. ^19^F NMR (376 MHz, acetone-*d*_6_) *δ* −105.50 ÷ −105.54 (m) ppm. HRMS (ESI, positive ion mode): calcd for C_16_H_15_BBrFN_3_O_4_SSi^+^ [M + H]^+^ 481.9808; found 481.9807.

#### 4.2.12 7-Fluoro-1,1-dimethyl-6-(1-(4-methylphenylsulfonyl)-1*H*-1,2,3-triazol-4-yl)benzo[*c*][1,2,5]oxasilaborol-3(1*H*)-ol (4c)

White powder, mp: 132–135 °C (dec.). Yield 0.32 g, (45%). ^1^H NMR (400 MHz, DMSO-*d*_6_) *δ* 9.09 (d, *J* = 2.8 Hz, 1H), 8.18 (t, *J* = 7.3 Hz, 1H), 8.12 (d, *J* = 8.5 Hz, 2H), 7.76 (dd, *J* = 7.5, 1.8 Hz, 1H), 7.56 (d, *J* = 8.3 Hz, 2H), 2.42 (s, 3H), 0.45 (s, 6H) ppm. ^11^B NMR (96 MHz, DMSO-*d*_6_) *δ* 29.7 ppm. ^19^F NMR (376 MHz, DMSO-*d*_6_) *δ* −104.30 (d, *J* = 7.0 Hz) ppm. HRMS (ESI, positive ion mode): calcd for C_17_H_18_BClFN_3_O_4_SSi^+^ [M + H]^+^ 418.0859; found 418.0859.

#### 4.2.13 7-Fluoro-1,1-dimethyl-6-(1-(2-fluorophenylsulfonyl)-1*H*-1,2,3-triazol-4-yl)benzo[*c*][1,2,5]oxasilaborol-3(1*H*)-ol (4d)

HRMS (ESI, positive ion mode): calcd for C_16_H_15_BF_2_N_3_O_4_SSi^+^ [M + H]^+^ 422.0608; found 422.0608.

#### 4.2.14 2,2′-(((1,1,3,3-Tetramethyldisiloxane-1,3-diyl)bis(2-fluoro-3,1-phenylene))bis(1*H*-1,2,3-triazole-4,1-diyl))bis(ethan-1-ol) (5a)

A glass vial was charged with CuSO_4_·5H_2_O (0.15 mmol, 0.1 equiv.), water (5 mL), THF (5 mL), 1c (1.5 mmol, 1 equiv.) and sodium ascorbate (0.15 mmol, 0.1 equiv.). The reaction mixture was cooled in an ice-water bath. Subsequently, 2-azidoethanol (1.5 mmol, 1 equiv.) was added. The reaction mixture was allowed to warm to room temperature and stirred overnight. Further workup was performed as described for 3a. The product was obtained as a white powder. Yield 0.16 g (38%). ^1^H NMR (400 MHz, CDCl_3_) *δ* 8.04 (td, *J* = 7.7, 1.8 Hz, 2H), 7.46 (d, *J* = 4.0 Hz, 2H), 7.43 (td, *J* = 5.1, 2.5 Hz, 2H), 7.24 (t, *J* = 7.7 Hz, 2H), 5.01 (broad s, 2H), 4.40–4.37 (m, 4H), 4.26–4.23 (m, 4H), 0.40 (s, 12H) ppm. ^13^C NMR (101 MHz, CDCl_3_) *δ* 163.3 (d, *J* = 245.5 Hz), 140.4 (d, *J* = 3.1 Hz), 135.0 (d, *J* = 11.9 Hz), 129.0 (d, *J* = 3.8 Hz), 125.5 (d, *J* = 31.4 Hz), 124.4 (d, *J* = 3.0 Hz), 124.0 (d, *J* = 14.7 Hz), 117.0 (d, *J* = 17.2 Hz), 61.2, 54.3, 1.0 (d, *J* = 2.1 Hz) ppm. ^19^F NMR (376 MHz, CDCl_3_) *δ* −101.65 ppm. calcd for C_24_H_31_F_2_N_6_O_3_Si_2_^+^ [M + H]^+^ 545.1959; found 545.1961.

#### 4.2.15 *N*,*N*′-((((1,1,3,3-Tetramethyldisiloxane-1,3-diyl)bis(2-fluoro-3,1-phenylene))bis(1*H*-1,2,3-triazole-4,1-diyl))bis(ethane-2,1-diyl))dibenzenesulfonamide (5b)

This compound was prepared as described for 5a using *N*-(2-azidoethyl)benzenesufonamide (1.5 mmol, 1 equiv.).^43^ It was isolated as a white solid. Yield 0.28 g (46%). ^1^H NMR (400 MHz, CDCl_3_) *δ* 8.24 (t, *J* = 7.4 Hz, 2H), 8.17 (broad s, 2H), 7.98 (d, *J* = 7.1 Hz, 1H), 7.58–7.42 (m, 10H), 7.27 (d, *J* = 8.1 Hz, 2H), 4.36 (s, 4H), 3.65 (s, 4H), 0.42 (s, 12H) ppm. ^1^H NMR (400 MHz, DMSO-*d*_6_) *δ* 8.34 (d, *J* = 3.9 Hz, 2H), 8.17 (td, *J* = 7.8, 1.8 Hz, 2H), 7.98 (t, *J* = 6.0 Hz, 2H), 7.77 (d, *J* = 6.8 Hz, 2H), 7.59–7.52 (m, 6H), 7.47 (t, *J* = 7.1 Hz, 2H), 7.32 (t, *J* = 7.4 Hz, 2H) 4.50 (t, *J* = 5.9 Hz, 4H), 3.30 (q, *J* = 6.0 Hz, 4H), 0.45 (s, 12H). ^19^F NMR (376 MHz, DMSO-*d*_6_) *δ* −98.34 ppm. ^13^C NMR (101 MHz, DMSO-*d*_6_) *δ* 162.2 (d, *J* = 242.6 Hz), 140.1, 139.6 (d, *J* = 2.9 Hz), 134.2 (d, *J* = 11.5 Hz), 132.4, 129.3, 129.2, 126.4, 125.3 (d, *J* = 30.8 Hz), 124.8 (d, *J* = 2.9 Hz), 124.3, 118.0 (d, *J* = 16.8 Hz), 49.4, 42.4, 1.2 ppm. calcd for C_36_H_41_F_2_N_8_O_5_S_2_Si_2_^+^ [M + H]^+^ 823.2142; found 823.2138.

### 4.3 Single crystal X-ray diffraction and structural analysis

Single crystals were grown by a solvent–evaporation method under air from the CHCl_3_ or acetone solutions. Selected crystals were measured at low temperature (100 K) with the use of Oxford Cryosystems nitrogen gas-flow device. The crystal structures were established in a conventional way *via* X-ray data refinement employing the Independent Atom Model (IAM). Data reduction and analysis were carried out with the CrysAlisPro suites of programs.^44^ All structures were solved by direct methods using SHELXS-97 (ref. 45) and refined using SHELXL-2016.^46^ All non-H atoms were refined anisotropically. All C–H atoms were placed in calculated positions with C–H distances of 0.95 Å and *U*_iso_(H) = 1.2*U*_eq_(C). The position H atoms of hydroxy groups were located from a difference electron density maps. The O–H distances were fixed to 0.84 Å with a standard deviation of 0.01 Å and the directionality of O–H was refined freely. The *U*_iso_ parameter was set to 1.5*U*_eq_ with respect to oxygen atoms. The crystal structure 1c contains large residual density peak (3.17 e Å^−3^), which cannot be reasonably explained by the mode. All-important crystallographic data including measurement, reduction, structure solution and refinement details are placed in Table S1 in ESI† or in the associated CIF files or can be retrieved from the Cambridge Crystallographic Data Centre as supplementary deposition no. 2304134 (1c), 2304135 (1c·MeCN), 2304136 (2c), 2304137 (3b), 2304138 (4d) and 2304139 (5b).

#### 4.3.1 Hirshfeld surface analysis

Analysis of Hirshfeld surfaces and their associated two-dimensional 2D-fingerprint plots was carried out with the CrystalExplorer (version 21.5) program.^47^ Graphical plots of the Hirshfeld surfaces mapped with normalized contact distance (*d*_norm_) use a red-white-blue color scale, where red indicates shorter contacts, white corresponds to contacts at the vdW distance, and blue is used for longer contacts. In addition, the Hirshfeld surfaces of all studied compounds were mapped with the values of electrostatic potential and fragment patch, the latter represents the fragment of the surface patches to adjacent molecules (Fig. S1.2 and S1.3, ESI†).

#### 4.3.2 Theoretical calculations

Theoretical calculations were performed at M06-2X[47]/6-311++G(d,p)^48,49^ level of theory using Gaussian16 program.^50^ The dimeric interaction energies were calculated starting from the geometries extracted from the crystal structures followed by the optimisation of positions of hydrogen atoms (positions of non-hydrogen atoms were retained from the crystal structure). The interaction energy was calculated using the counterpoise procedure, which includes the correction for basis set superposition error (BSSE). The topological analysis of the electron density was prepared using QTAIM approach^51^ and it was carried out with AIMAll package.^52^ The wavefunction was generated at M06-2X/6-311++G(d,p) level of theory. In the framework of this approach, bond baths (BP) and bond critical points (BCP) were identified. The non-covalent interaction index (NCI) analysis^53^ was performed with AIMAll. The natural bond orbital (NBO)^54^ analysis was calculated at the same level of theory. The localized orbitals were visualized using Avogadro software.^55^

### 4.4 Antimicrobial activity

#### 4.4.1 Bacterial and fungal strains and their growth conditions

Direct antimicrobial activity was determined against the following standard strains: (1) Gram-positive cocci: methicillin-sensitive *Staphylococcus aureus* ATCC 6538P (MSSA), methicillin-resistant *S. aureus* subsp. *aureus* ATCC 43300 (MRSA), *S. epidermidis* ATCC 12228, *Enterococcus faecium* ATCC 6057, *E. faecalis* ATCC 29212, *Bacillus subtilis* ATCC 6633; (2) Gram-negative bacteria from *Enterobacteriales* order: *Escherichia coli* ATCC 25922, *Klebsiella pneumoniae* ATCC 13883, *Proteus mirabilis* ATCC 12453, *Enterobacter cloacae* DSM 6234, *Serratia marcescens* ATCC 13880; (3) Gram-negative non-fermentative rods: *Acinetobacter baumannii* ATCC 19606, *Pseudomonas aeruginosa* ATCC 27853, *Stenotrophomonas maltophilia* ATCC 12714, *S. maltophilia* ATCC 13637, *Burkholderia cepacia* ATCC 25416, *Bordetella bronchiseptica* ATCC 4617; (4) yeasts: *Candida albicans* ATCC 90028, *C. parapsilosis* ATCC 22019, *C. krusei* ATCC 6258, *C. tropicalis* (Castellani) Berkhout ATCC 750, *C. tropicalis* IBA 171, *C. guilliermondii* IBA 155, and *Saccharomyces cerevisiae* ATCC 9763. All strains were stored at ÷80 °C. Prior to testing, each bacterial strain was subcultured twice on tryptic soy agar TSA (bioMerieux) medium and yeast strains on Sabouraud dextrose agar (bioMerieux) for 24–48 h at 30 °C to ensure viability.

#### 4.4.2 Determination of antimicrobial activity

The direct antimicrobial activity against yeast, Gram-positive and Gram-negative bacterial strains was evaluated as previously described^17,18^ by the disc-diffusion method and the MIC determination assays according to the EUCAST^56^ and CLSI^40,57*a*^ recommendations. Bactericidal (MBC) activity was performed according to the CLSI recommendations.^57*b*^ Each 0.02 mL bacterial culture samples from clear wells with the MIC and above the MIC values were transferred onto TSA agar plates (bioMerieux) and incubated for 24–48 h at 37 °C. The MFC was defined as the lowest concentration of a agent that kills 99.9% of the initial inoculum of yeast and was evaluated after establishing the MIC values according to the EUCAST recommendation.^56*b*^ Each 0.1 mL yeast culture samples from clear wells with the MIC and above the MIC values were transferred onto Sabouraud dextrose agar plates (bioMerieux) and incubated for 24–48 h at 30 °C. The following reference agents were utilized: fluconazole (in the case of yeast), linezolid (for Gram-positive bacteria), and nitrofurantoin (for Gram-negative rods). The new compounds were dissolved in DMSO (Sigma). In the disc-diffusion method, paper discs containing 0.4 mg of the tested new compounds per disc were used. The MIC and MBC/MFC values were determined up to 400 mg L^−1^.

#### 4.4.3 Determination of MICs in the presence of PAβN

To determine the ability of the Gram-negative bacterial strains to remove new tested compounds by MDR efflux pumps, the MIC values of these agents, with or without the pump inhibitor, PAβN (20 mg L^−1^) (Sigma) were evaluated as previously described.^17,18^ To minimize the influence of PAβN on the destabilization of bacterial cell covers, the tests were conducted in the presence of 1 mM MgSO_4_ (Sigma).^58^ At least a 4-fold reduction in the MIC value after the addition of PAβN was considered significant.^17,18^

## Author contributions

S. L. and A. E. L.: conceptualization of the paper and supervision of the research; S. L.: funding acquisition, P. P.: synthesis and compound characterization, K. D. and K. W.: single-crystal X-ray diffraction; K. D.: theoretical calculations; J. K.: antimicrobial activity studies; K. D., A. E. L., and S. L.: data analysis; P. P., K. D., J. K., A. E. L. and S. L.: writing the original draft. All authors have read and agreed to the published version of the manuscript.

## Conflicts of interest

There are no conflicts to declare.

## Supplementary Material

RA-014-D4RA02137A-s001

RA-014-D4RA02137A-s002
