# Supplementary material for: Ethynyl-substituted benzosiloxaboroles: the role of C(π)⋯B interactions in their crystal packing and use in Cu(i)-catalyzed 1,3-dipolar cycloaddition
Source: RSC Adv. 2024 May 17;14(23):16069–82. doi: 10.1039/d4ra02137a (PMC11099988; doi:10.1039/d4ra02137a)
Supplement: RA-014-D4RA02137A-s001 [file RA-014-D4RA02137A-s001.pdf]

Electronic Supplementary Material (ESI) for RSC Advances.
This journal is © The Royal Society of Chemistry 2024

Supporting Information

Ethynyl-substituted benzosiloxaboroles: the role of C(π)…B interactions in their crystal packing and use in Huisgen 1,3-dipolar cycloaddition

P. Pacholak,[a,b] K. Durka*,[a] K. Woźniak[b] J. Krajewska,[c] A. E. Laudy*,[c] S. Luliński*[a]

[a] *Faculty of Chemistry, Warsaw University of Technology, Noakowskiego 3, 00-664 Warsaw, Poland*

[b] *University of Warsaw, Faculty of Chemistry, Warsaw, Poland, Pasteura 1, 02-093*

[c] *Department of Pharmaceutical Microbiology and Bioanalysis, Medical University of Warsaw, Banacha 1b, 02-097 Warsaw, Poland*

Email address: sergiusz.lulinski@pw.edu.pl (S. Luliński).

Email address: alaudy@wp.pl (A. E. Laudy).

1. Structural and computational studies

Table S1.1. Selected crystal data, data collection and refinement parameters for **1c**, **1c·MeCN** and **2c**.

	**1c**	**1c·MeCN**	**2c**
Formula	$C_{10}H_{10}BO_2SiF$	$C_{12}H_{13}BO_2SiNF$	$C_{10}H_{10}O_2ClSiB$
Weight	220.08	261.13	236.53
T / K	100(2)	100(2)	100(2)
Crystal system	triclinic	monoclinic	triclinic
Space group	P-1	$P2_1/m$	P-1
a /Å	6.9862(2)	8.6127(2)	6.9876(3)
b / Å	7.4451(2)	7.0177(2)	7.7137(4)
c / Å	10.5854(2)	11.1617(3)	10.6833(5)
α / °	92.553(2)	90	85.701(4)
β / °	97.855(2)	97.153(2)	81.858(4)
γ / °	91.766(2)	90	89.401(4)
V / Å^3	544.49(2)	669.38(3)	568.42(5)
Z	2	2	2
ρ_{calc} / g·cm^{-3}	1.342	1.296	1.382
μ / mm^{-1}	0.204	0.179	3.788
F(000)	228.0	272.0	244.0
Crystal size / mm^3	0.342 × 0.129 × 0.089	0.212 × 0.174 × 0.074	0.12 × 0.11 × 0.10
Radiation	MoKα (λ = 0.71073)	MoKα (λ = 0.71073)	Cu Kα (λ = 1.54184)
2Θ range for data collection / °	3.888 to 57.656	4.766 to 67.666	8.384 to 154.988
Index ranges	$-9 \leq h \leq 9$, $-10 \leq k \leq 9$, $-14 \leq l \leq 14$	$-13 \leq h \leq 12$, $-10 \leq k \leq 10$, $-16 \leq l \leq 17$	$-8 \leq h \leq 8$, $-9 \leq k \leq 9$, $-13 \leq l \leq 13$
Reflections collected	24998	14332	5118
Independent reflections	2683 [R_{int} = 0.0255, R_{sigma} = 0.0128]	2733 [R_{int} = 0.0292, R_{sigma} = 0.0228]	2404 [R_{int} = 0.0154, R_{sigma} = 0.0148]
Data/restraints/parameters	2683/0/139	2733/1/114	2404/1/141
Goodness-of-fit	1.046	1.085	1.084
Final R indexes [I>=2σ (I)]	R_1 = 0.0284, wR_2 = 0.0768	R_1 = 0.0335, wR_2 = 0.0916	R_1 = 0.0300, wR_2 = 0.0773
Final R indexes [all data]	R_1 = 0.0304, wR_2 = 0.0783	R_1 = 0.0402, wR_2 = 0.0971	R_1 = 0.0302, wR_2 = 0.0775
Largest diff. peak/hole / e·Å^{-3}	0.44/–0.23	0.47/–0.34	0.38/–0.37

Table S1.2. Selected crystal data, data collection and refinement parameters for **3b**, **4d** and **5b**.

	**3b**	**4d**	**5b**
Formula	$C_{16}H_{16}BN_3O_5SiFClS$	$C_{17}H_{15}N_3O_4SiSBF_2Cl_3$	$C_{36}H_{40}N_8O_5F_2Si_2S_2$
Weight	455.73	540.63	823.06
T / K	100(2)	100(2)	100(2)
Crystal system	monoclinic	triclinic	monoclinic
Space group	$P2/c$	P-1	$P2_1/c$
a /Å	15.7378(4)	8.8484(4)	12.2547(2)
b / Å	5.96710(10)	11.1678(4)	21.3124(4)
c / Å	21.2618(5)	12.0146(5)	14.7890(2)
α / °	90	89.089(3)	90
β / °	97.318(2)	77.254(3)	91.9810(10)
γ / °	90	80.690(3)	90
V / Å^3	1980.41(8)	1142.50(8)	3860.24(11)
Z	4	2	4
ρ_{calc} / g·cm^{-3}	1.528	1.572	1.416
μ / mm^{-1}	3.677	0.592	2.388
F(000)	936.0	548.0	1720.0
Crystal size / mm^3	0.15 × 0.11 × 0.10	0.21 × 0.107 × 0.104	0.231 × 0.146 × 0.087
Radiation	CuKα (λ = 1.54178)	MoKα (λ = 0.71073)	CuKα (λ = 1.54178)
2Θ range for data collection / °	5.662 to 145.38	4.784 to 60.008	7.218 to 153.4
Index ranges	$-19 \le h \le 19$, $-7 \le k \le 7$, $-21 \le l \le 26$	$-11 \le h \le 12$, $-15 \le k \le 15$, $-16 \le l \le 16$	$-15 \le h \le 15$, $-26 \le k \le 26$, $-18 \le l \le 12$
Reflections collected	15611	19660	22446
Independent reflections	3890 [R_{int} = 0.0270, R_{sigma} = 0.0222]	6007 [R_{int} = 0.0317, R_{sigma} = 0.0475]	8052 [R_{int} = 0.0357, R_{sigma} = 0.0380]
Data/restraints/parameters	3890/1/265	6007/1/313	8052/0/496
Goodness-of-fit	1.040	1.035	1.017
Final R indexes [I>=2σ (I)]	R_1 = 0.0298, wR_2 = 0.0764	R_1 = 0.0489, wR_2 = 0.1085	R_1 = 0.0445, wR_2 = 0.1170
Final R indexes [all data]	R_1 = 0.0360, wR_2 = 0.0810	R_1 = 0.0670, wR_2 = 0.1181	R_1 = 0.0497, wR_2 = 0.1226
Largest diff. peak/hole / e·Å^{-3}	0.35/–0.37	0.74/–0.68	0.56/–0.36

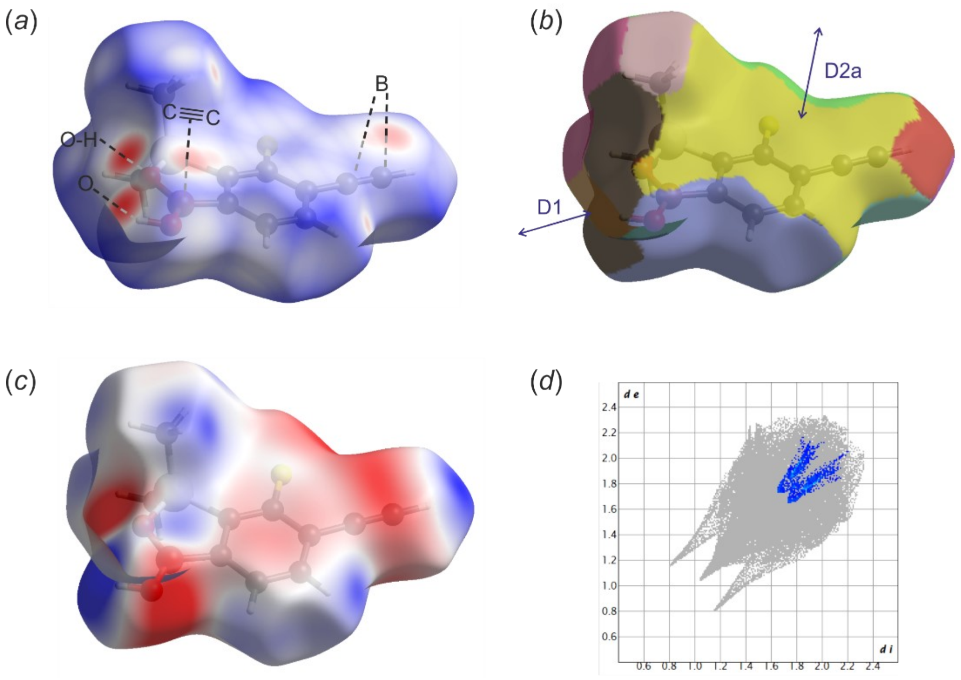

Figure S1.1 Hirshfeld surfaces generated for **1c** with mapped (*a*) d_{norm} property value over the range –0.50 to 1.30, (*b*) fragment patch, (*c*) electrostatic potential over the range –0.06 to 0.08 a.u. (*d*) Fingerprint plots with marked C…B contacts.

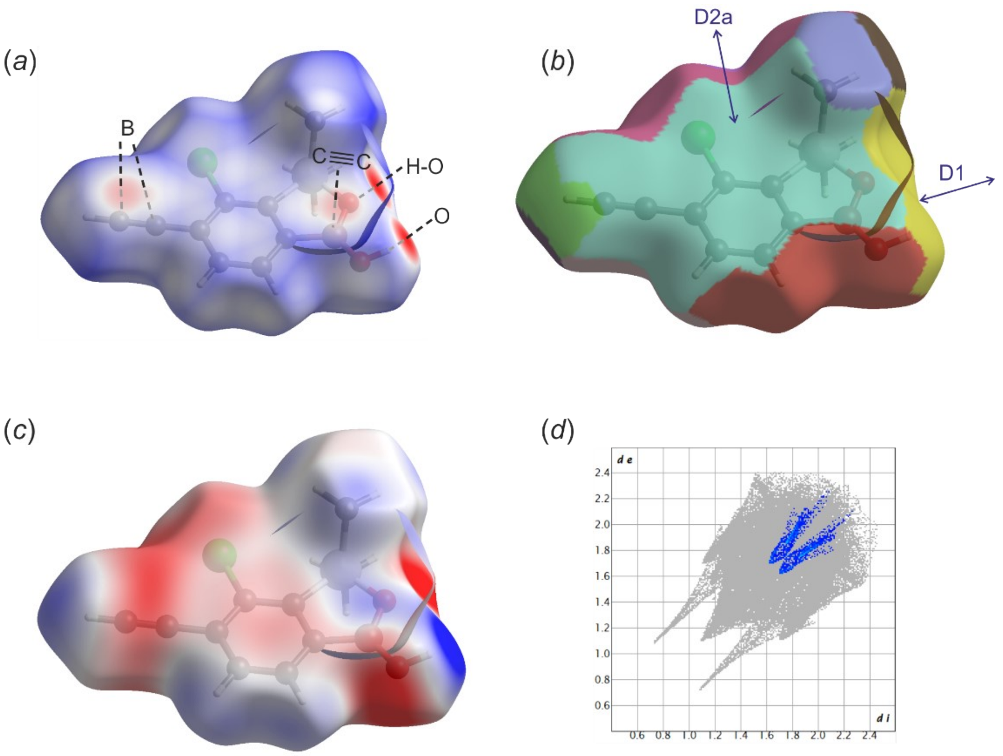

Figure S1.2. Hirshfeld surfaces generated for **2c** with mapped (*a*) d_{norm} property value over the range –0.50 to 1.30, (*b*) fragment patch, (*c*) electrostatic potential over the range –0.06 to 0.08 a.u. (*d*) Fingerprint plots with marked C…B contacts.

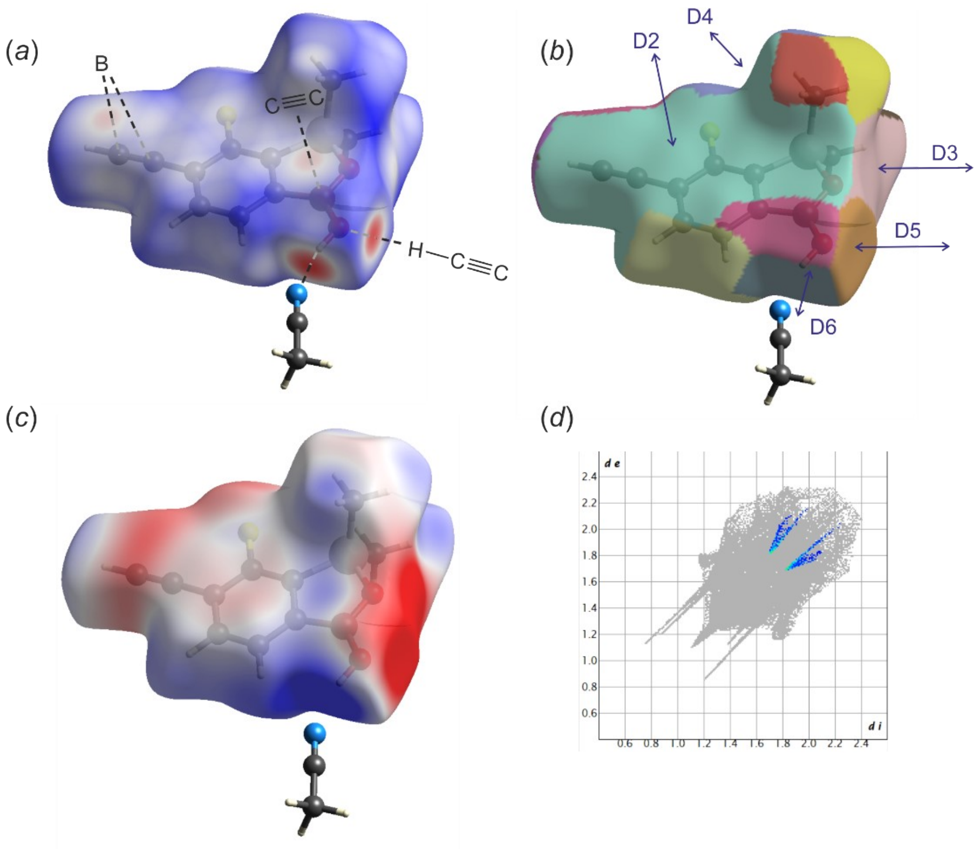

Figure S1.3. Hirshfeld surfaces generated for **1c**·MeCN with mapped (*a*) d_{norm} property value over the range –0.50 to 1.30, (*b*) fragment patch, (*c*) electrostatic potential over the range –0.06 to 0.08 a.u. (*d*) Fingerprint plots with marked C…B contacts.

Table S1.3. The intermolecular donor-acceptor orbital C(π)…B interaction energies (kJ·mol^{-1}) estimated by 2nd-order perturbation theory within NBO analysis.

Structure	**1c**		**2c**		**1c**·MeCN
Motif	D2a	D2b	D2a	D2b	D2
$E(\pi_{CC(ethynyl)} \rightarrow p_B)$	5.1	2.6	5.4	2.3	2.7
$E(\pi_{CC(aromatic)} \rightarrow \pi^*_{CC(ethynyl)})$	0.6	0.8	0.6	1.0	0.5

Table S1.4. Electron density (ρ, e·Å^{-3}) and negative Laplacian ($\nabla^2\rho$, e·Å^{-5}) at BCPs of C(π)…B interactions.

Structure	**1c**		**2c**		**1c**·MeCN
Motif	D2a	D2b	D2a	D2b	D2
ρ	0.033	0.035	0.035	0.036	0.036
$\nabla^2\rho$	0.33	0.36	0.35	0.36	0.35

2. Antimicrobial activity.

Table S2.1. The antibacterial activity of tested agents against standard Gram-positive strains

**Agent tested**	**MIC in mg·L^{-1} [MBC in mg·L^{-1}][a] (Diameter of inhibition zone in mm)**					
	S. aureus **ATCC 6538P**	***S. aureus*** **ATCC 43300 MRSA**	***S. epidermidis*** **ATCC 12228**	***E. faecalis*** **ATCC 29212**	***E. faecium*** **ATCC 6057**	***B. subtilis*** **ATCC 6633**[b]
**1c**	25 *[50/400]*[c] (25)	25 [50] (20)	**12.5** *[25/>400]*[c] (29)	100 (15)	100 (16)	NT (26)
**2c**	**12.5** *[25/200]*[c] (21)	**12.5** [25] (23)	**12.5** [400] (26)	50 (20)	50 (21)	NT (27)
**3a**	25 (22)	50 (20)	50 (24)	200 (-)	200 (11)	NT (21)
**3b**	50 (20)	50 (22)	50 (23)	200 (-)	200 (-)	NT (21)
**3c**	**12.5** *[25/>400]*[c] (22)	**12.5** [25] (23)	**12.5** *[25/>400]*[c] (29)	100 (16)	50 (16)	NT (27)
**4a**	25 (20)	50 (20)	50 (25)	200 (-)	200 (-)	NT (23)
**4b**	25 (25)	50 (21)	50 (20)	200 (-)	200 (-)	NT (18)
**4c**	25 (20)	50 (22)	50 (24)	200 (-)	200 (-)	NT (20)
**5a**	>400 (-)	>400 (-)	>400 (-)	>400 (-)	>400 (-)	NT (-)
**5b**	>400 (-)	>400 (-)	>400 (-)	>400 (-)	>400 (-)	NT (-)
**LIN**[d]	1 [>128] (25)	2 [>128] (25)	1 [>128] (26)	2 [>128] (15)	2 [>128] (14)	NT (30)

The highest activity against Gram-positive bacteria indicated by the low MIC values (≤12.5 mg·L^{-1}) is shown in boldface.
(-): The inhibition zone was not observed in the disc-diffusion method. The diameter of the paper discs was 9 mm.
[a] Only the MBC values ≤400 mg·L^{-1}are presented.
[b] The growth type of *B. subtilis* in the MHB medium prevented reading the MIC values of tested substances.
[c] The Eagle effect [1,2] was observed during the determination of the MBC value of the same tested agents against *Staphylococcus* spp. strains. The Eagle effect is shown in the italic face.
[d] LIN, linezolid was used as a reference agent active against Gram-positive bacteria. The diameter of a commercial disc containing 0.03 mg of linezolid was 6 mm; the MIC of linezolid was determined according to the CLSI recommendations [3].

Table S2.2. The antibacterial activity of tested agents against standard Gram-negative strains.

Agent tested	MIC in mg·L^{-1} [MBC in mg·L^{-1}][a] / x-fold reduction of MIC in the presence of PAßN[b] (Diameter of inhibition zone in mm)										
	***E. coli* ATCC 25922**	***K. pneumoniae* ATCC 13883**	***P. mirabilis* ATCC 12453**	***E. cloacae* DSM 6234**	***S. marcescens* ATCC 13880**	***A. baumannii* ATCC 19606**	***P. aeruginosa* ATCC 27853**	***S. maltophilia* ATCC 13637**	***S. maltophilia* ATCC 12714**	***B. cepacia* ATCC 25416[c]**	***B. bronchiseptica* ATCC 4617[c]**
**1c**	400 [400]/**4** (13)	400 [400]/2 (-)	400/2 (-)	400/2 (-)	>400/**8** (-)	>400 (-)	>400 (-)	200 [400]/2 (11)	100 [400] (12)	>400 (-)	400 (-)
**2c**	>400/**8** (-)	>400 (-)	>400/2 (-)	>400/2 (-)	>400/**4** (-)	>400/**4** (-)	>400 (-)	400 [400]/**8** (13)	400/**4** (-)	>400 (-)	400 (-)
**3a**	>400 (-)	>400 (-)	>400 (-)	>400 (-)	>400 (-)	>400 (-)	>400 (-)	>400 (-)	>400 (-)	>400 (-)	>400 (-)
**3b**	>400 (-)	>400 (-)	>400 (-)	>400 (-)	>400 (-)	>400 (-)	>400 (-)	>400/2 (-)	>400 (-)	>400 (-)	400 (-)
**3c**	>400 (-)	>400 (-)	>400 (-)	>400 (-)	>400 (-)	>400 (-)	>400 (-)	400/**4** (15)	>400/**4** (-)	>400 (-)	>400 (-)
**4a**	>400 (-)	>400 (-)	>400 (-)	>400 (-)	>400 (-)	>400 (-)	>400 (-)	>400 (-)	>400 (-)	>400 (-)	>400 (-)
**4b**	>400 (-)	>400 (-)	>400 (-)	>400 (-)	>400 (-)	>400 (-)	>400 (-)	>400/2 (-)	>400/2 (-)	>400 (-)	400 (-)
**4c**	>400 (-)	>400 (-)	>400 (-)	>400 (-)	>400 (-)	>400 (-)	>400 (-)	>400/2 (-)	>400 (-)	>400 (-)	>400 (-)
**5a**	>400 (-)	>400 (-)	>400 (-)	>400 (-)	>400 (-)	>400 (-)	>400 (-)	>400 (-)	>400 (-)	>400 (-)	>400 (-)
**5b**	>400 (-)	>400 (-)	>400 (-)	>400 (-)	>400 (-)	>400 (-)	>400 (-)	>400 (-)	>400 (-)	>400 (-)	>400 (-)
**Nf[g]**	8 [8] (24)	32 [32] (23)	128 [>128] (9)	32 [32] (17)	128 [>128] (12)	64 [128] (9)	>128 [>128] (-)	128 [>128] (-)	128 [>128] (-)	32 [32] (12)	64 [128] (-)

PAβN: efflux pump inhibitor. The significant decreases (at least a 4-fold) in the MIC values of tested compounds after the addition of PAβN are shown in boldface. The test was performed in the MHB medium supplemented with 1 mM $MgSO_4$.

(-): The inhibition zone was not observed in the disc-diffusion method. The diameter of the paper discs was 9 mm.

[a] Only the MBC values ≤400 mg·L^{-1} are presented.

[b] In the table, only at least 2-fold decreases in the MIC values of tested compounds after the addition of PAβN are presented.

[c] The growth of *B. cepacia* ATCC 25416 and *B. bronchiseptica* ATCC 4617 strains was inhibited in the MHB medium supplemented with 1 mM $MgSO_4$ and 20 mg·L^{-1} PAβN.

[d] Nf, nitrofurantoin was used as a reference agent active against Gram-negative bacteria. The diameter of a commercial disc containing 0.3 mg of nitrofurantoin was 6 mm; the MIC of nitrofurantoin was determined according to the CLSI recommendations [3].

Table S2.3. The antifungal activity of tested agents against standard yeasts strains.

Agent tested	MIC in mg·L^{-1} [MFC in mg·L^{-1}][a] (Diameter of inhibition zone in mm)						
	C. albicans **ATCC 90028**	***C. parapsilosis*** **ATCC 22019**	***C. tropicalis*** **ATCC 750**	***C. tropicalis*** **IBA 171**	***C. guilliermondii*** **IBA 155**	***C. krusei*** **ATCC 6258**	***S. cerevisiae*** **ATCC 9763**
**1c**	200 (20)	400 (15)	100 (20)	**25** [400] (24)	400 (23)	400 (23)	200 (14)
**2c**	100 [400] (22)	200 (21)	50 [400] (20)	**12.5** [200] (25)	100 (37)	100 (15)	**12.5** [200] (28)
**3a**	>400 (-)	>400 (-)	>400 (-)	>400 (-)	400 (-)	>400 (-)	>400 (-)
**3b**	>400 (-)	>400 (-)	>400 (-)	>400 (-)	>400 (-)	>400 (-)	>400 (-)
**3c**	>400 (-)	>400 (-)	>400 (-)	>400 (-)	>400 (-)	>400 (-)	>400 (-)
**4a**	>400 (-)	>400 (-)	>400 (-)	>400 (-)	>400 (-)	>400 (-)	>400 (-)
**4b**	>400 (-)	>400 (-)	>400 (-)	>400 (-)	>400 (-)	>400 (-)	>400 (-)
**4c**	>400 (-)	>400 (-)	>400 (-)	>400 (-)	>400 (-)	>400 (-)	>400 (-)
**5a**	>400 (-)	>400 (-)	>400 (-)	>400 (-)	>400 (-)	>400 (-)	>400 (-)
**5b**	>400 (-)	>400 (-)	>400 (-)	>400 (-)	>400 (-)	>400 (-)	>400 (-)
**Fl**[b]	1 (43)	2 (32)	0.38 (40)	0.38 (39)	0.75 (40)	64[c] (16)	16[d] (12)

The highest activity against yeasts indicated by the low MIC values (25 mg·L^{-1}) is shown in boldface.
(-): The inhibition zone was not observed in the disc-diffusion method. The diameter of the paper discs was 9 mm.
[a] Only the MFC values ≤400 mg·L^{-1} are presented.
[b] FL, fluconazole was used as a reference antifungal agent; the diameter of a commercial disc containing 0.025 mg of fluconazole was 6 mm; the MIC value of fluconazole was determined by the Etest method [4].
[c] The ellipse was visible pointing the MIC value 64 mg·L^{-1}. However, with macro-colonies up to a concentration ≥256 mg·L^{-1}. In accordance with the recommendations for the Etest method, the MIC value of fluconazole against *C. krusei* can also be interpreted as ≥256 mg·L^{-1} [4,5]. *C. krusei* is intrinsically resistant to fluconazole.
[d] The ellipse was visible pointing the MIC value 16 mg·L^{-1}, with colonies up to concentration ≥256 mg·L^{-1}. There are no recommendations for the Etest method interpretation of the MIC value of fluconazole against *S. cerevisiae*. The obtained MIC 16 mg·L^{-1} is in line with the published results [6].

3. References

1. H. Eagle and A. D. Musselman. The rate of bactericidal action of penicillin in vitro as a function of its concentration, and its paradoxically reduced activity at high concentrations against certain organisms. *J. Exp. Med.*, 1948, **88**, 99–131.

2. A. Prasetyoputri, A. M. Jarrad, M. A. Cooper and M. A. T. Blaskovich. The Eagle Effect and Antibiotic-Induced Persistence: Two Sides of the Same Coin? *Trends Microbiol.*, 2019, **27**, 339–354.

3. Clinical and Laboratory Standards Institute. 2012. Methods for dilution antimicrobial susceptibility tests for bacteria that grow aerobically. Approved Standard, 9th ed. CLSI guideline M07-A9. Wayne, PA, USA.

4. ETEST. Application guide, BioMerieux., https://www.biomerieux-usa.com/sites/subsidiary_us/files/supplementary_inserts_-_16273_-_b_-_en_-_eag_-_etest_application_guide-3.pdf (accessed 1 February 2024).

5. A. Espinel-Ingroff. Etest for antifungal susceptibility testing of yeasts, *Diagn. Microbiol. Infect. Dis.*, 1994, **19**, 217–220.

6. M. A. Pfaller, M. Bale, B. Buschelman, M. Lancaster, A. Espinel-Ingroff, J. H. Rex and M. G. Rinaldi. Selection of candidate quality control isolates and tentative quality control ranges for *in vitro* susceptibility testing of yeast isolates by National Committee for Clinical Laboratory Standards proposed standard methods, *J. Clin. Microbiol.*, 1994, **32**, 1650–1653.

4. NMR spectra of new compounds

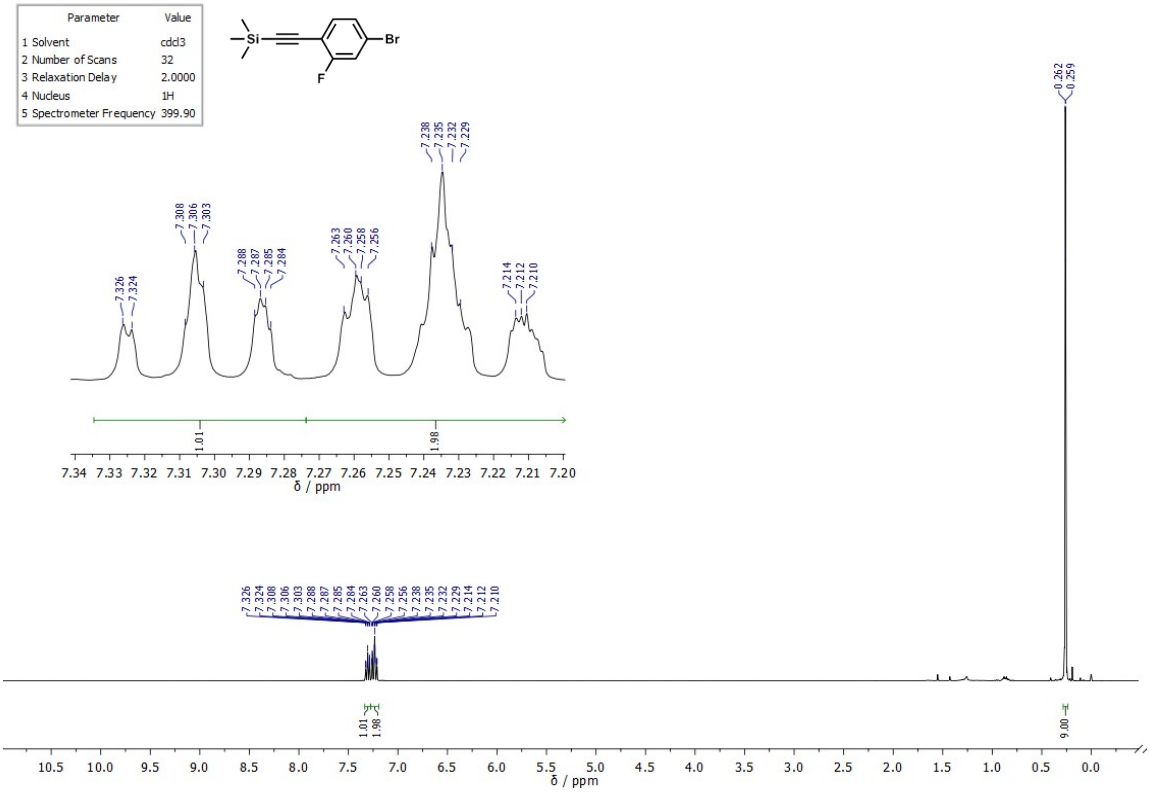

Figure S4.1. ^{1}H NMR (400 MHz, $CDCl_3$) spectrum of **1a**.

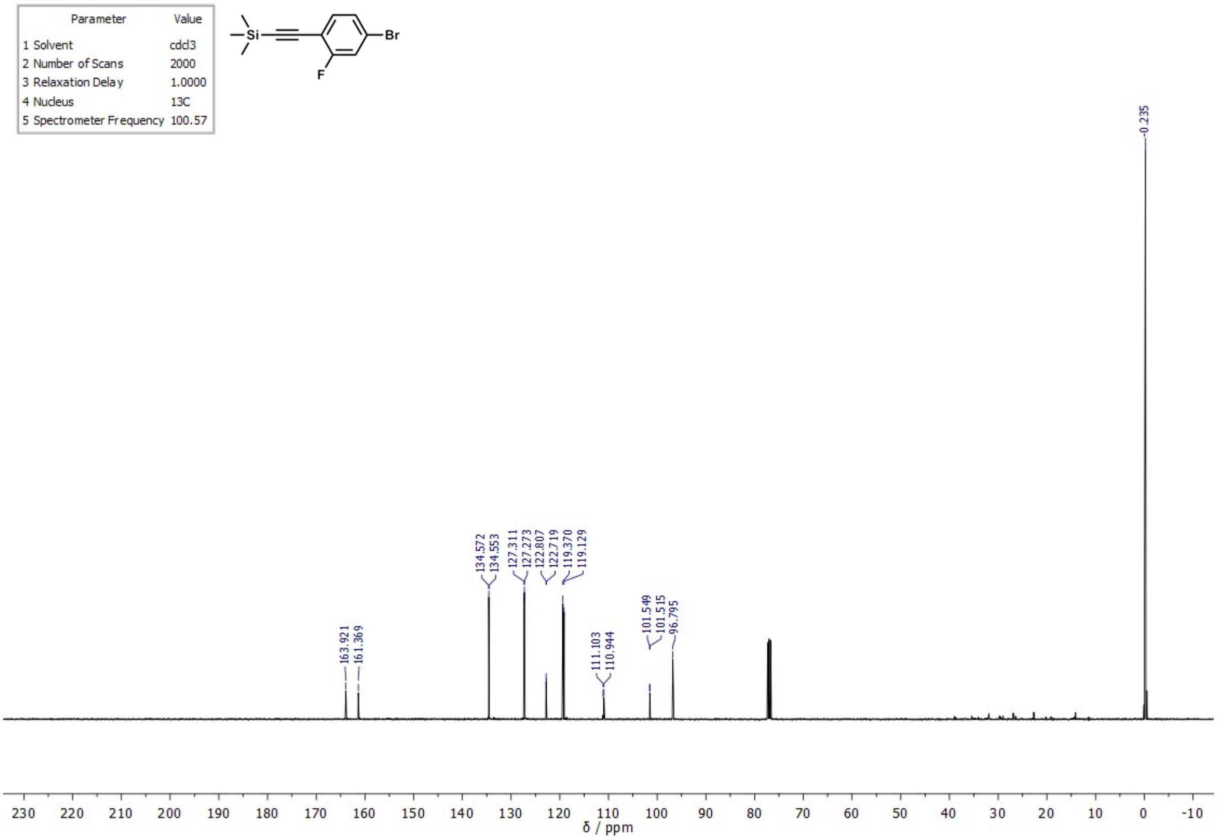

Figure S4.2. ^{13}C NMR (101 MHz, $CDCl_3$) spectrum of **1a**.

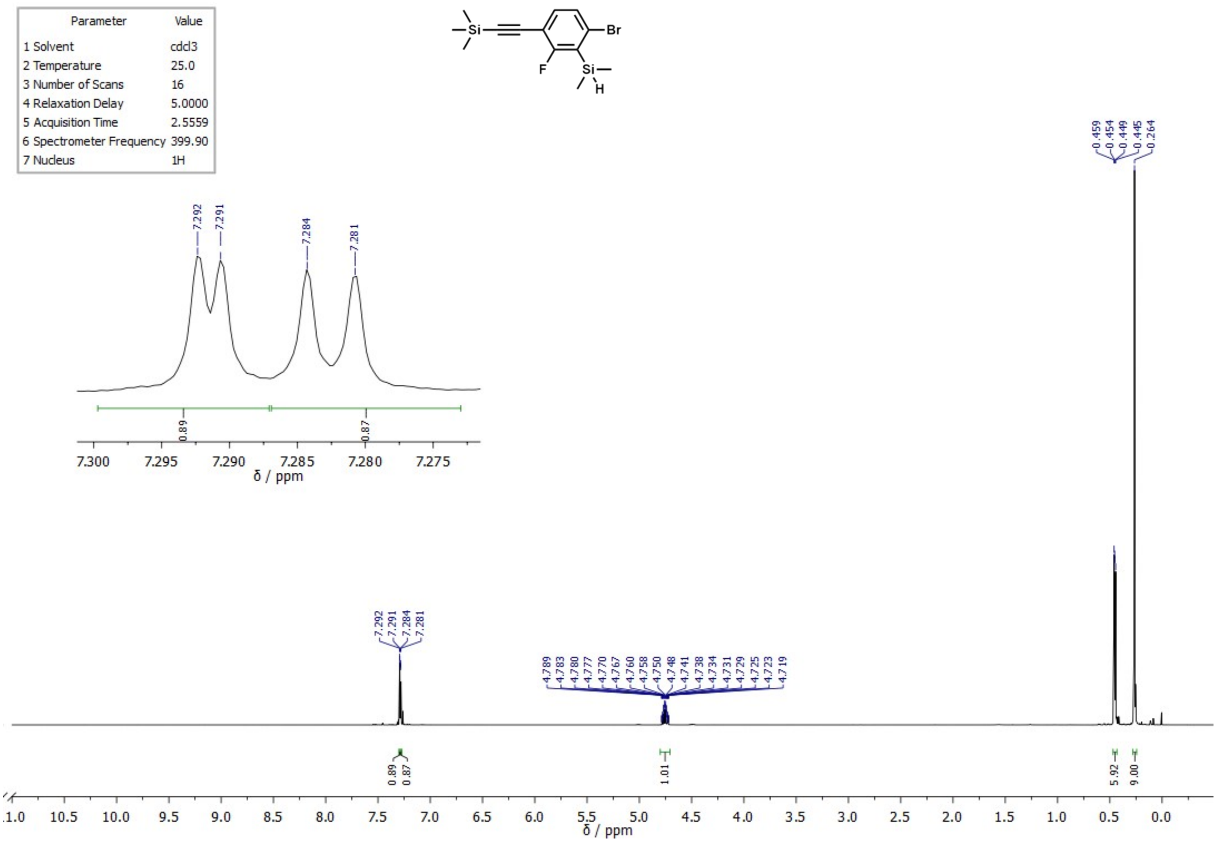

Figure S4.3. ^{1}H NMR (400 MHz, $CDCl_3$) spectrum of **1b**.

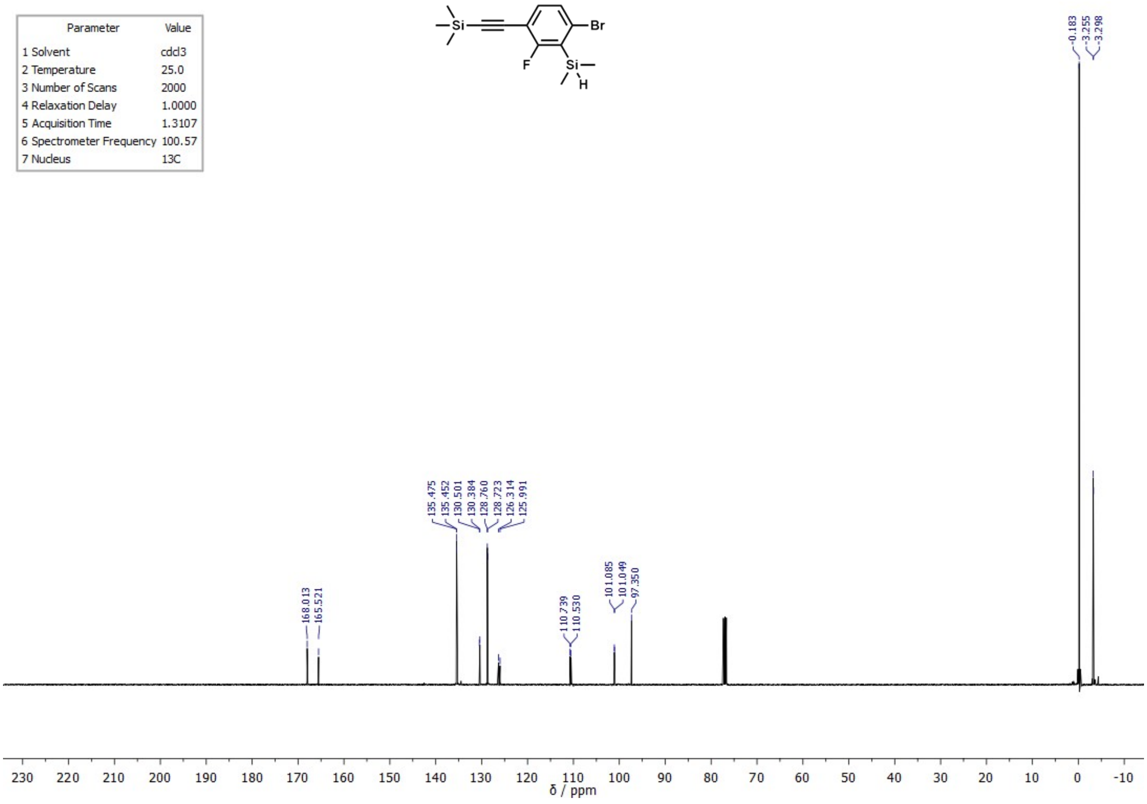

Figure S4.4. ^{13}C NMR (101 MHz, $CDCl_3$) spectrum of **1b**.

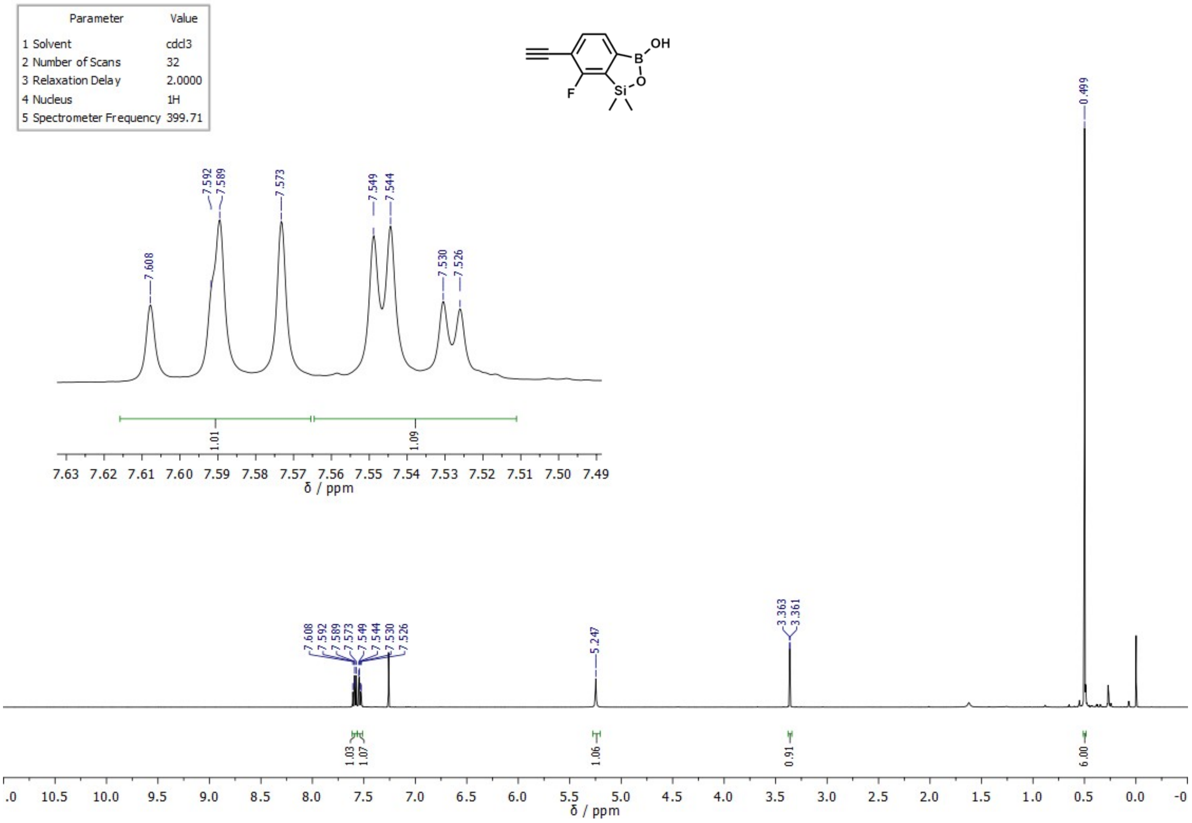

Figure S4.5. ^{1}H NMR (400 MHz, $CDCl_3$) spectrum of **1c**.

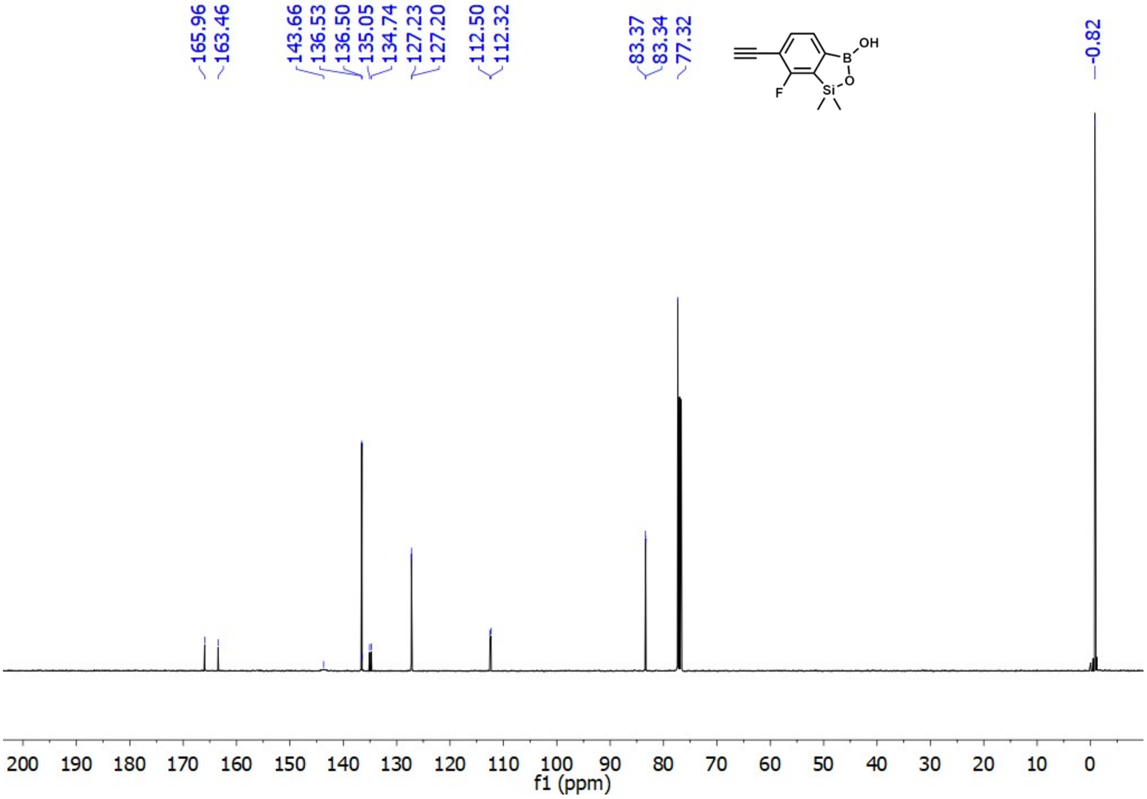

Figure S4.6. ^{13}C NMR (101 MHz, $CDCl_3$) spectrum of **1c**.

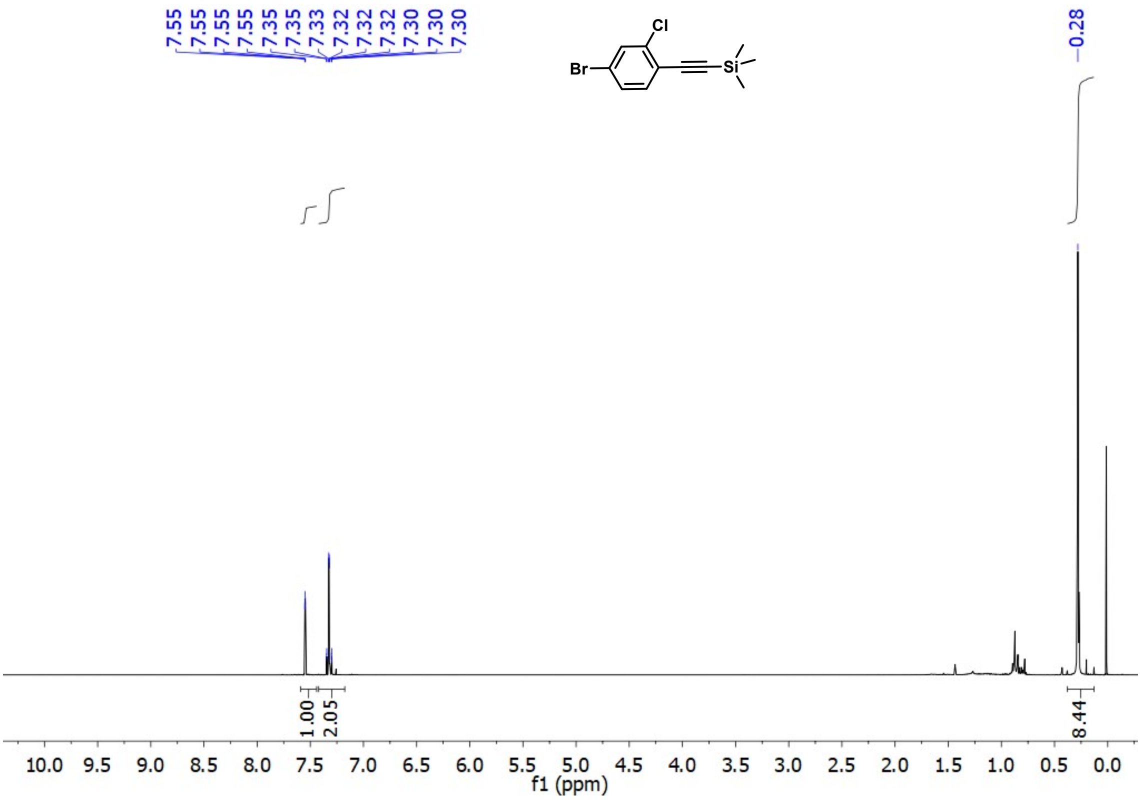

Figure S4.7. ^{1}H NMR (400 MHz, $CDCl_3$) spectrum of **2a**.

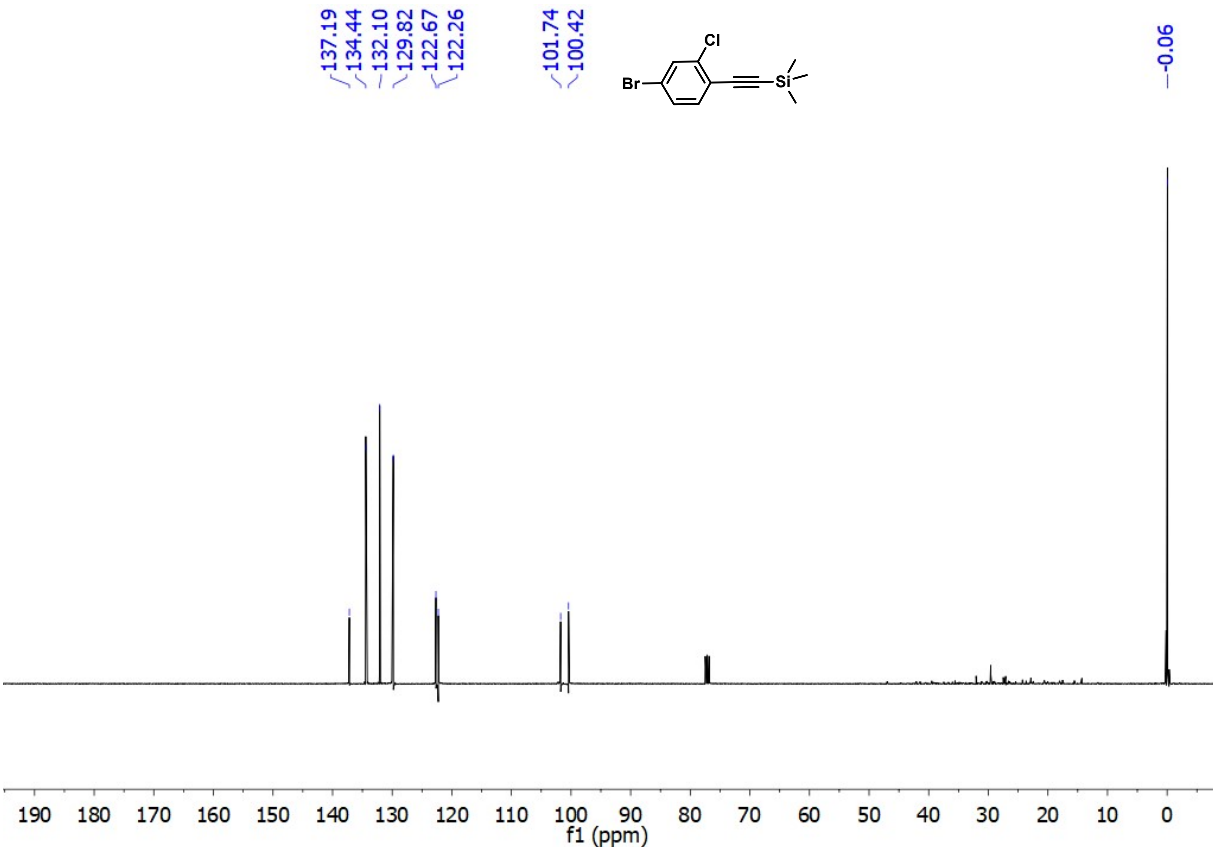

Figure S4.8. ^{13}C NMR (101 MHz, $CDCl_3$) spectrum of **2a**.

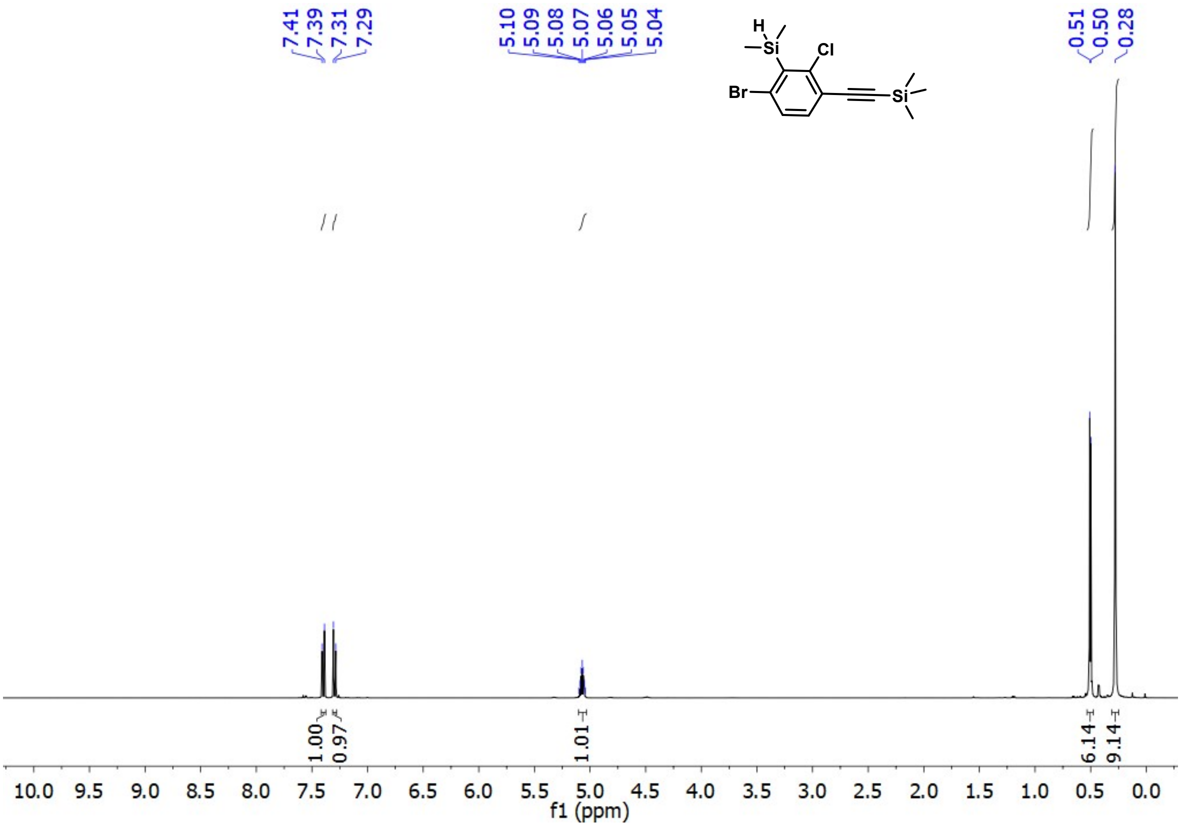

Figure S4.9. ^{1}H NMR (400 MHz, $CDCl_3$) spectrum of **2b**.

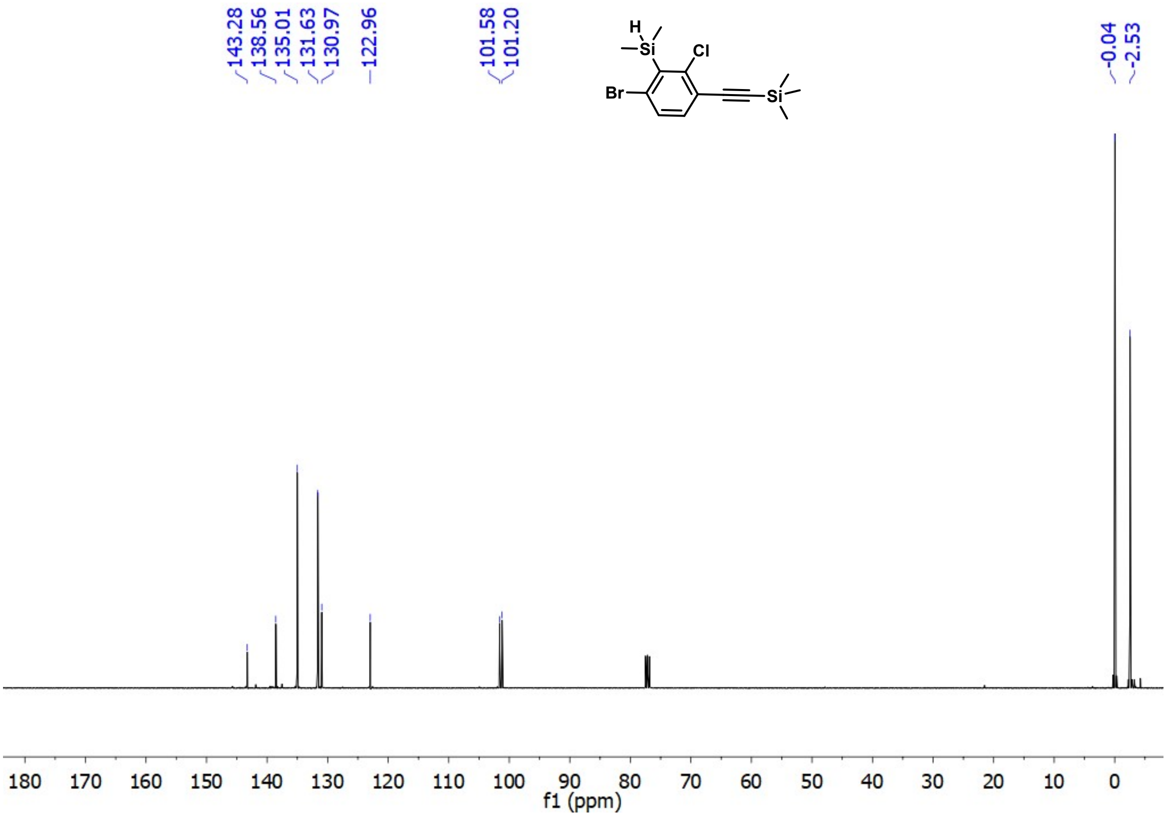

Figure S4.10. ^{13}C NMR (101 MHz, $CDCl_3$) spectrum of **2b**.

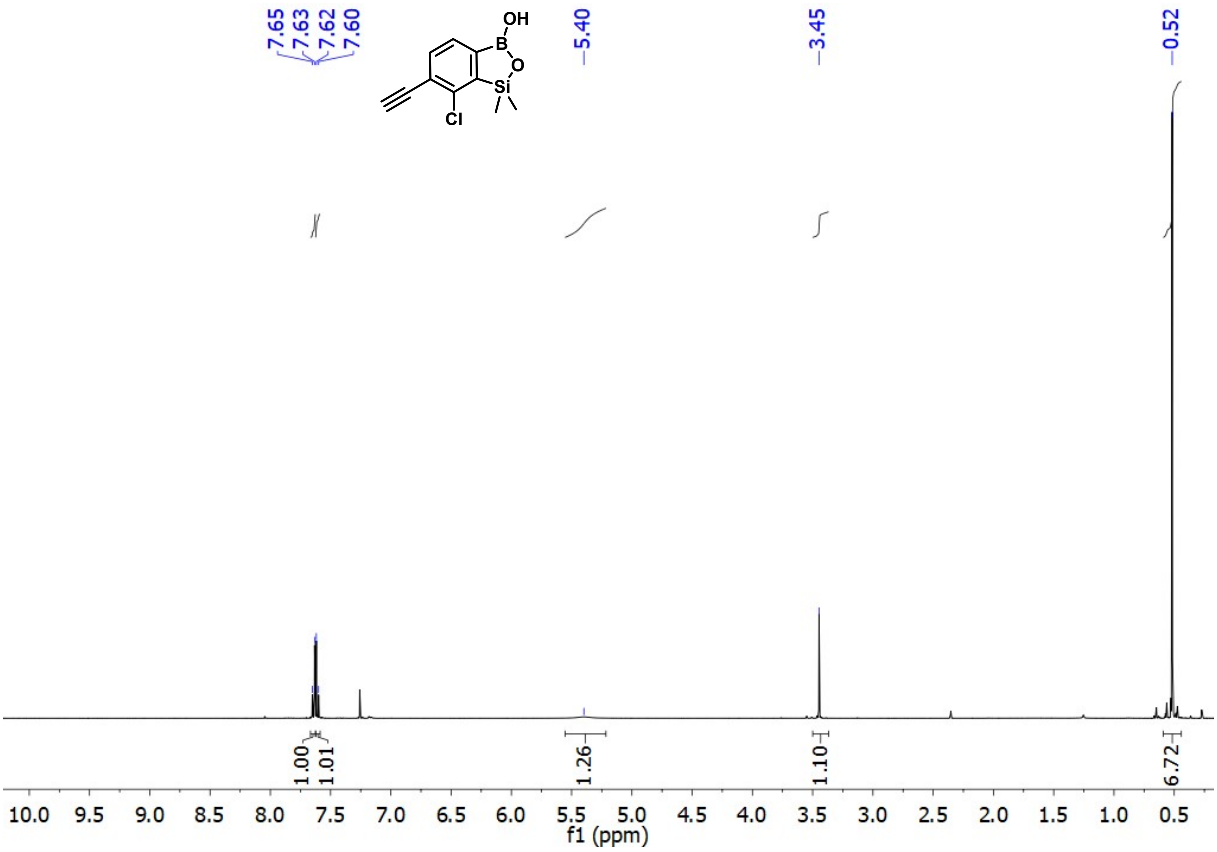

Figure S4.11. ^{1}H NMR (400 MHz, $CDCl_3$) spectrum of **2c**.

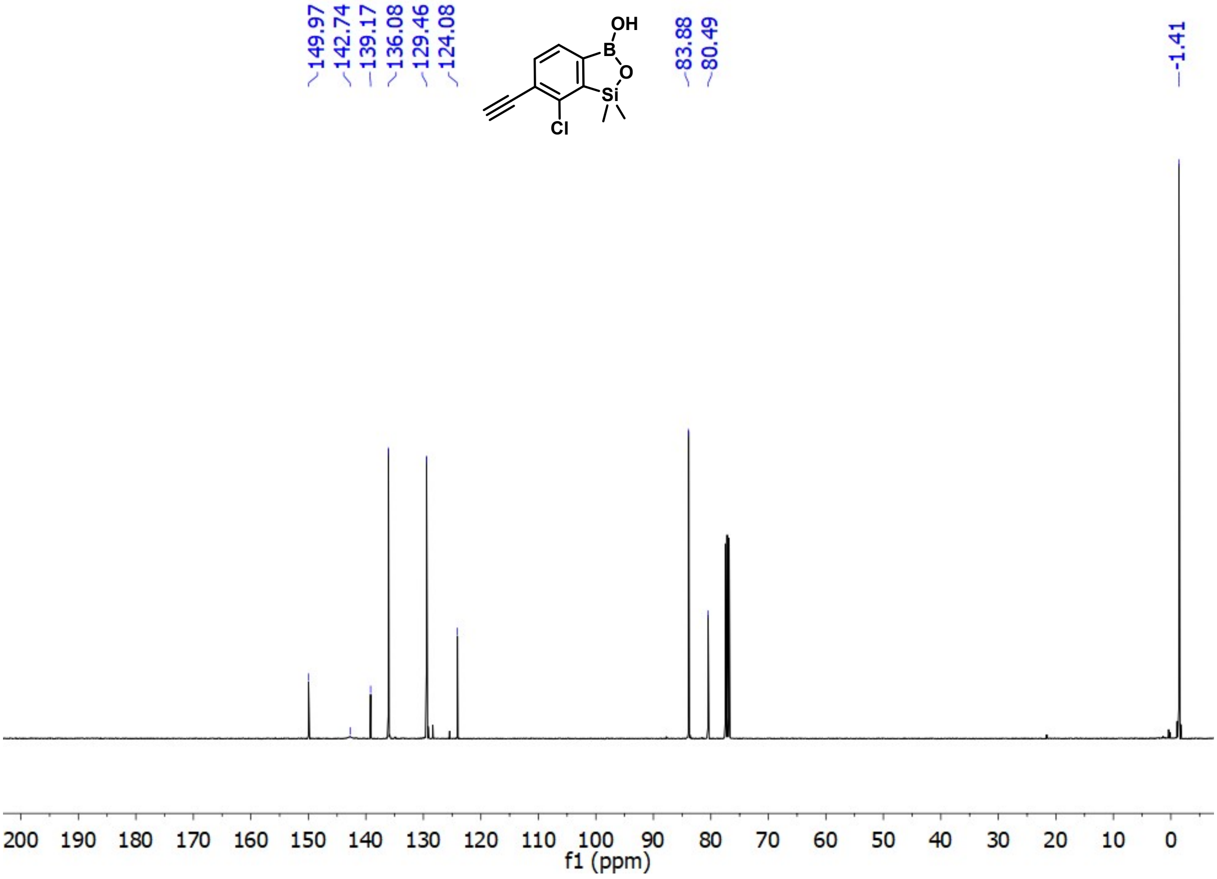

Figure S4.12. ^{13}C NMR (101 MHz, $CDCl_3$) spectrum of **2c**.

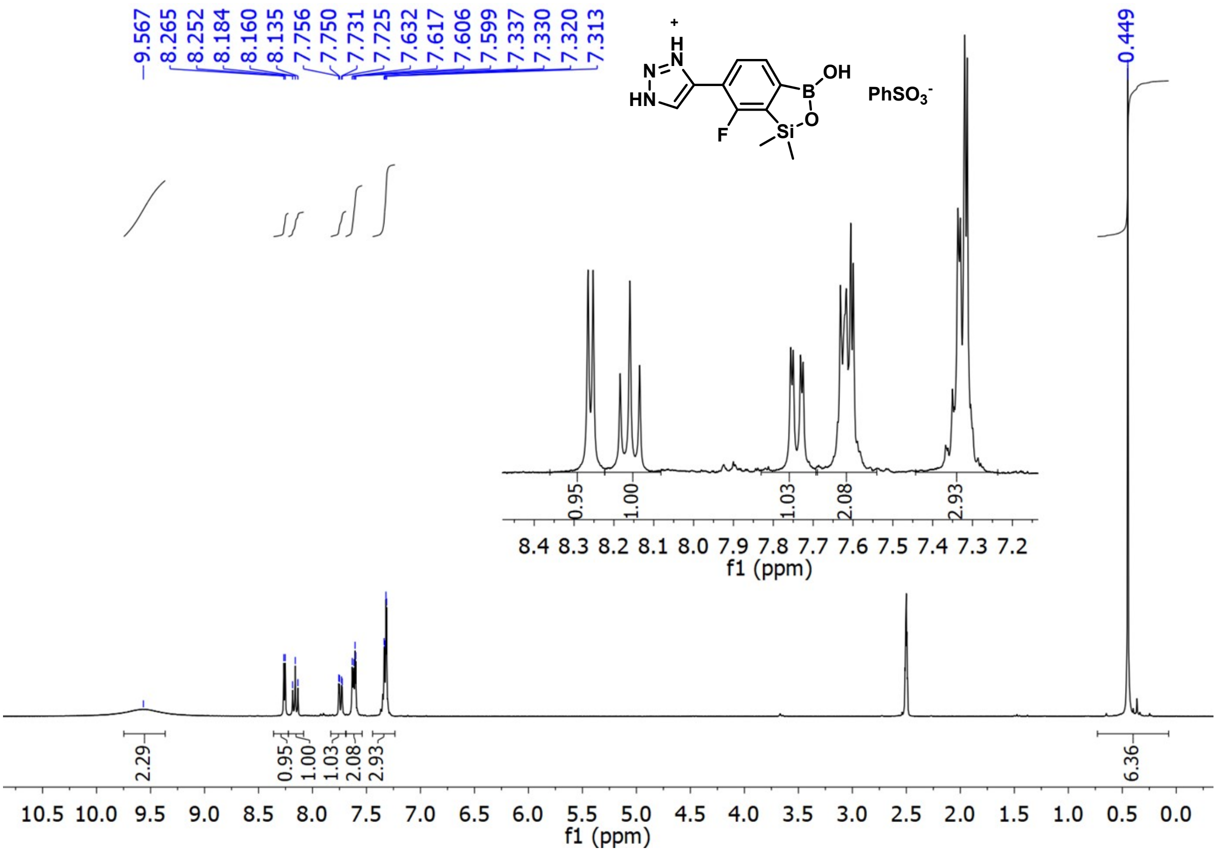

Figure S4.13. ^{1}H NMR (400 MHz, DMSO-d_6) spectrum of **3a**.

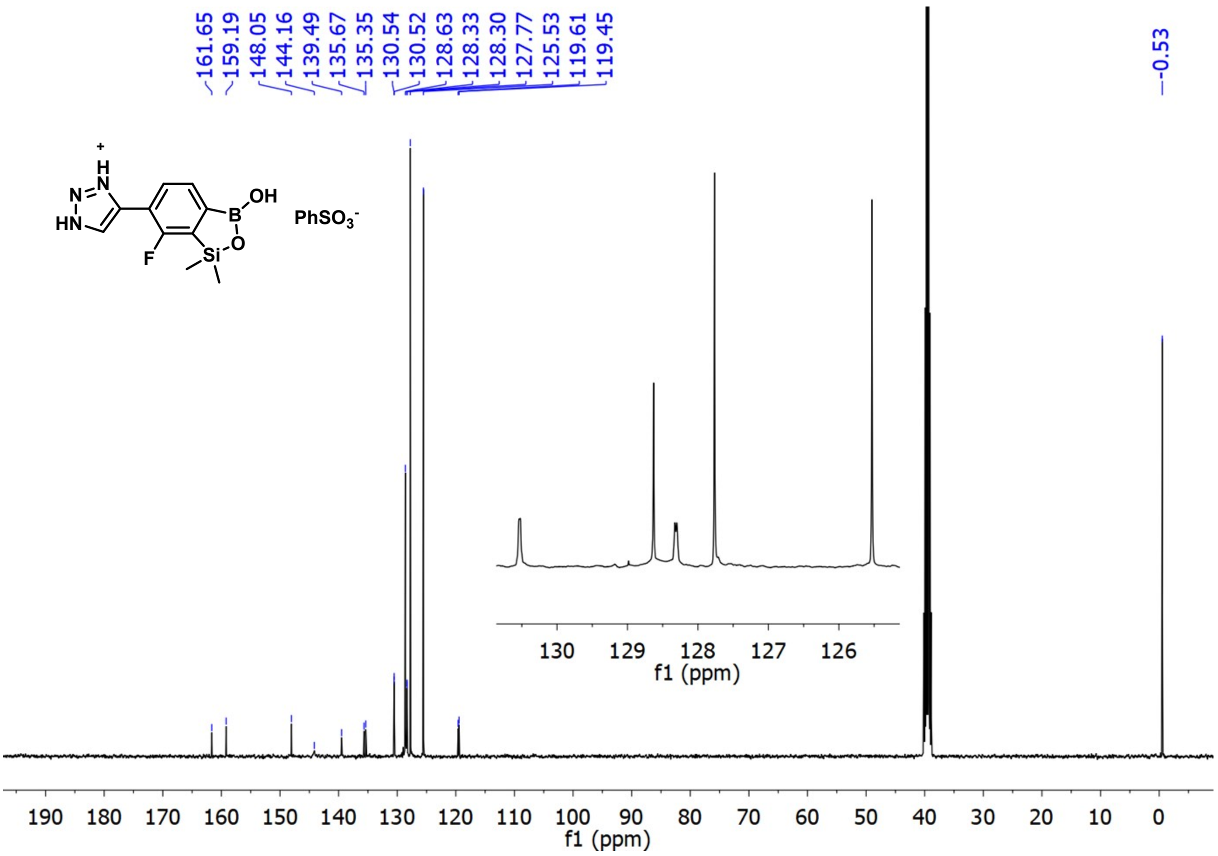

Figure S4.14. ^{13}C NMR (101 MHz, DMSO-d_6) spectrum of **3a**.

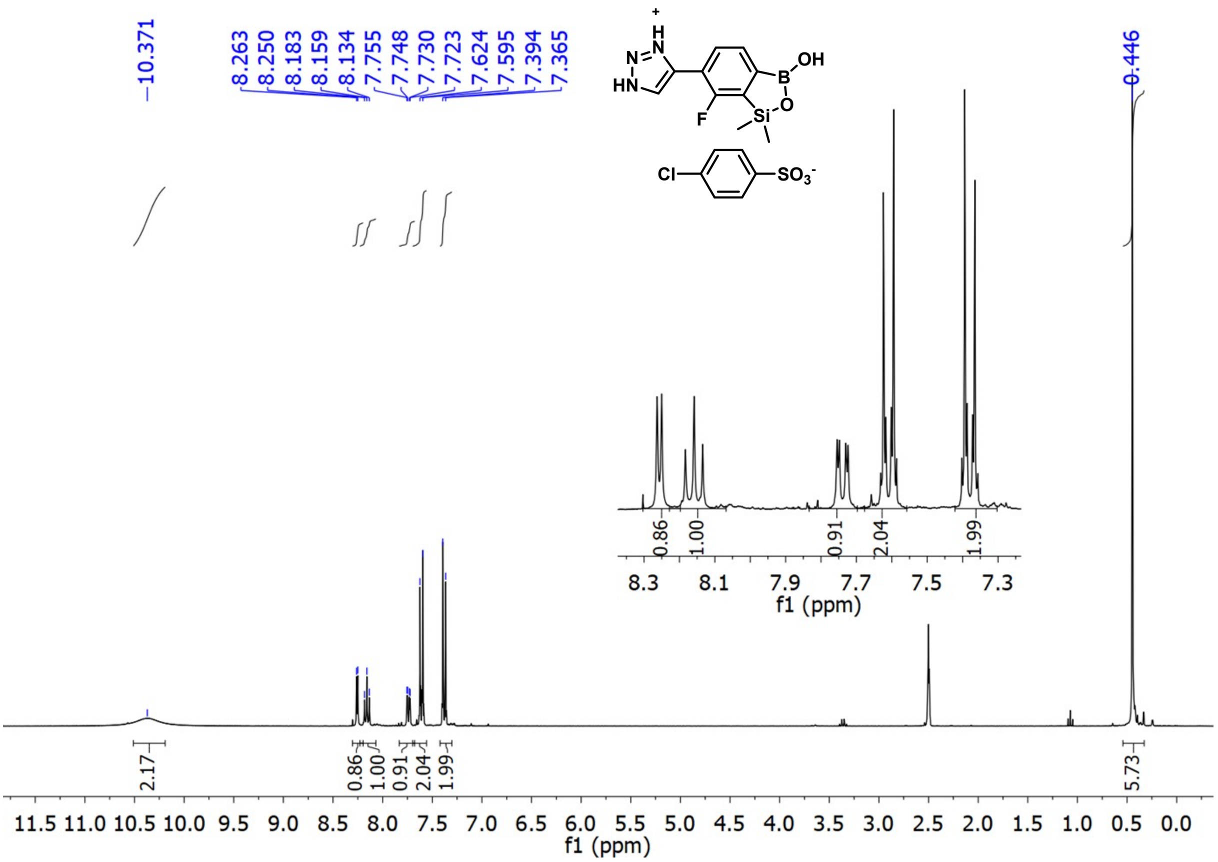

Figure S4.15. ^{1}H NMR (400 MHz, DMSO-d_6) spectrum of **3b**.

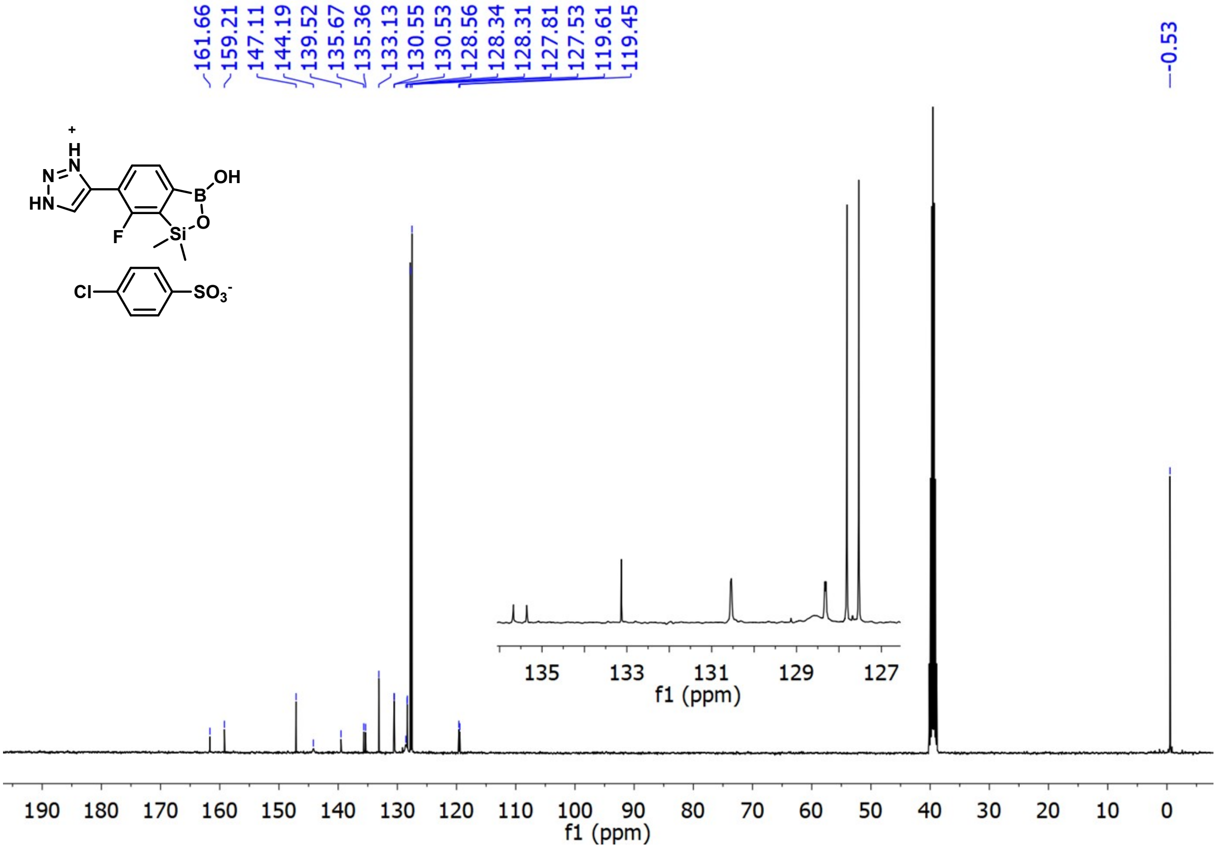

Figure S4.16. ^{13}C NMR (101 MHz, DMSO-d_6) spectrum of **3b**.

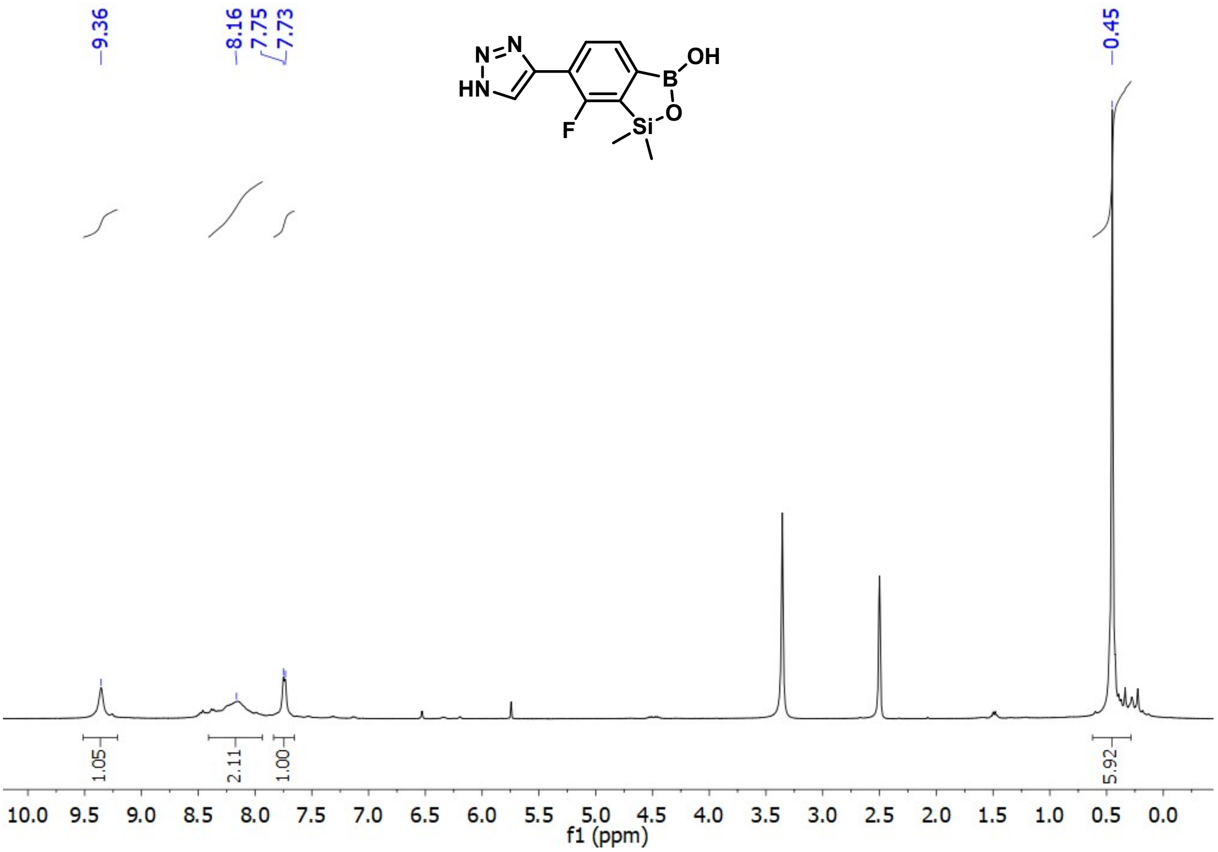

Figure S4.17. ^{1}H NMR (400 MHz, DMSO-d_6) spectrum of **3c**.

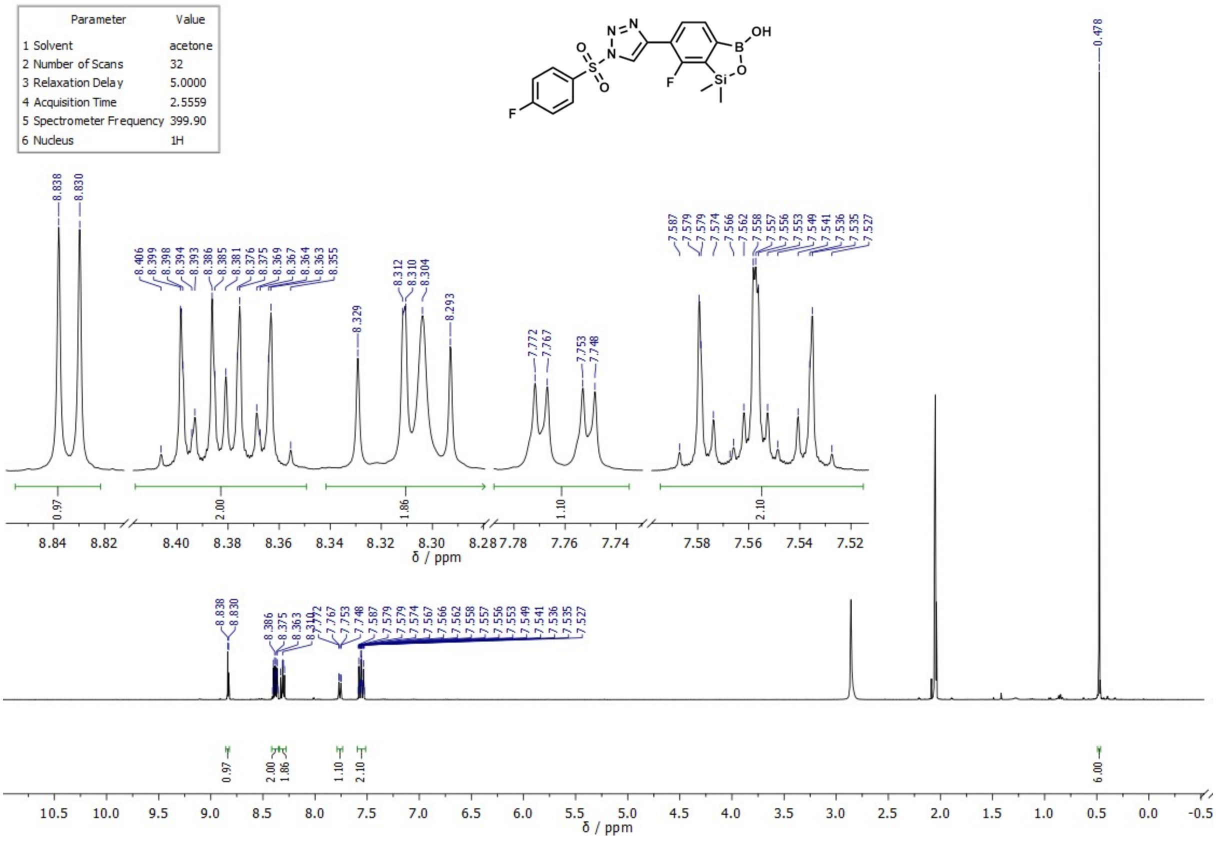

Figure S4.18. ^{1}H NMR (400 MHz, acetone-d_6) spectrum of **4a**.

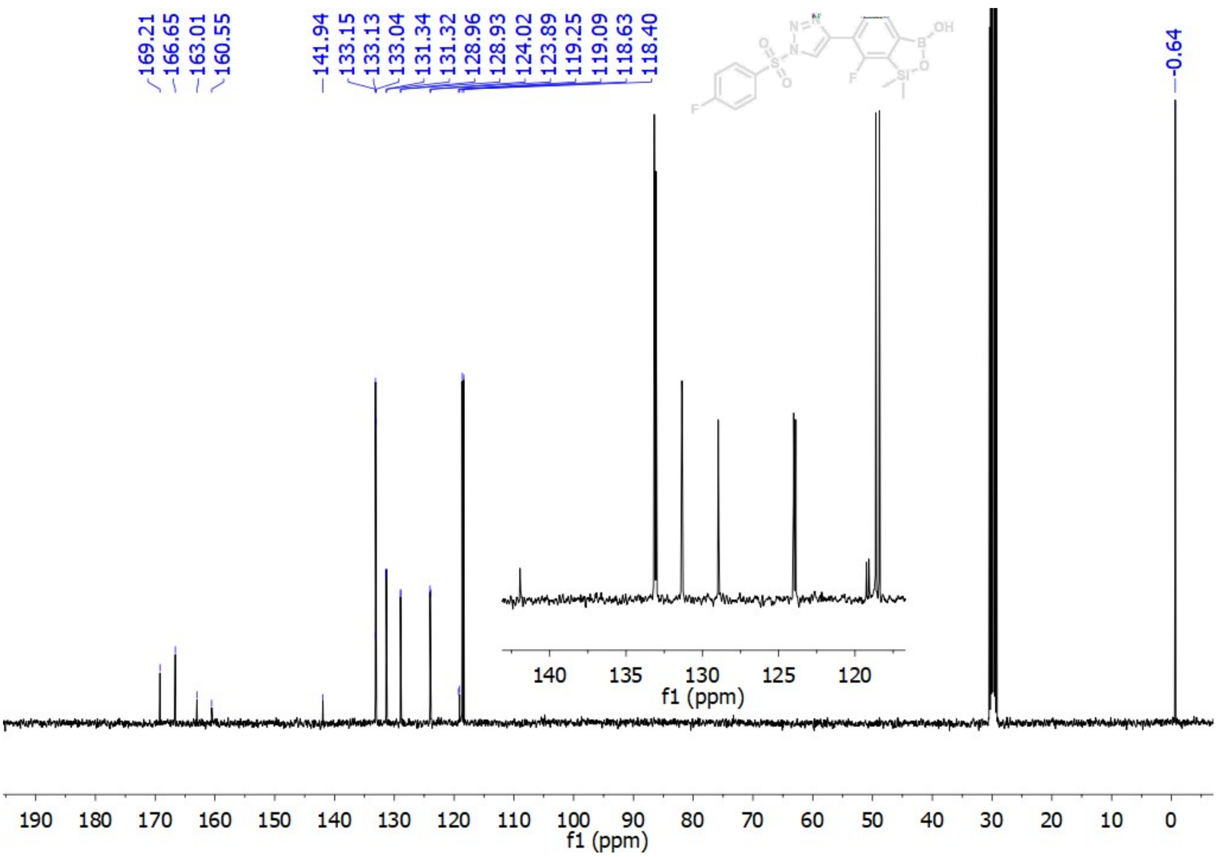

Figure S4.19. ^{13}C NMR (400 MHz, acetone-d_6) spectrum of **4a**.

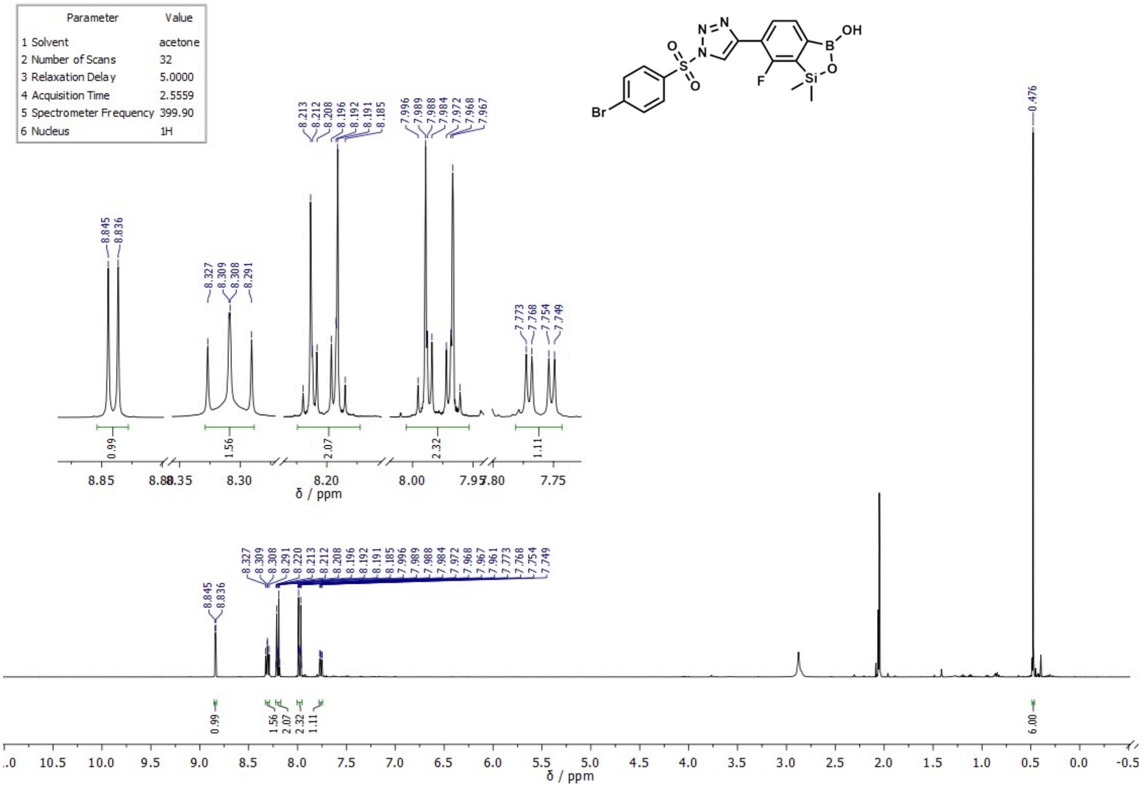

Figure S4.20. ^{1}H NMR (400 MHz, acetone-d_6) spectrum of **4b**.

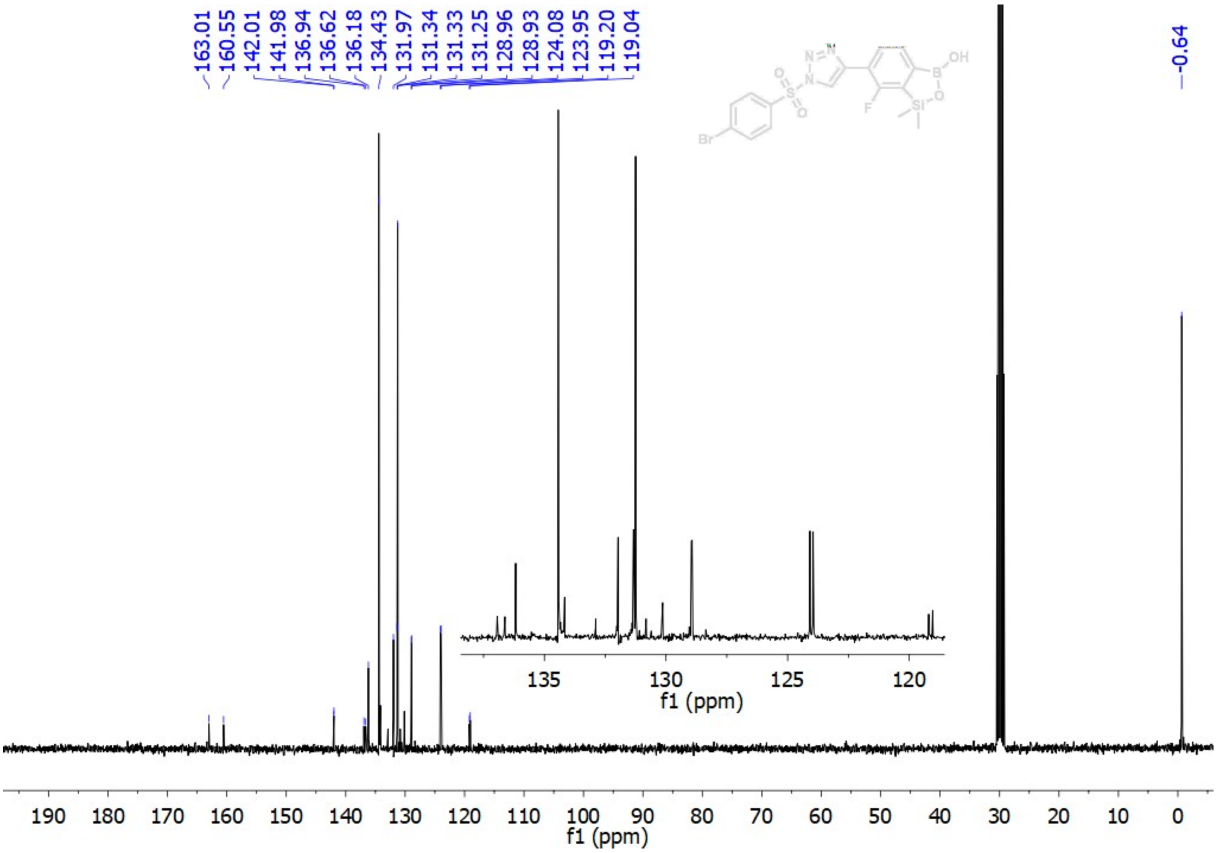

Figure S4.21. ^{13}C NMR (400 MHz, acetone-d_6) spectrum of **4b**.

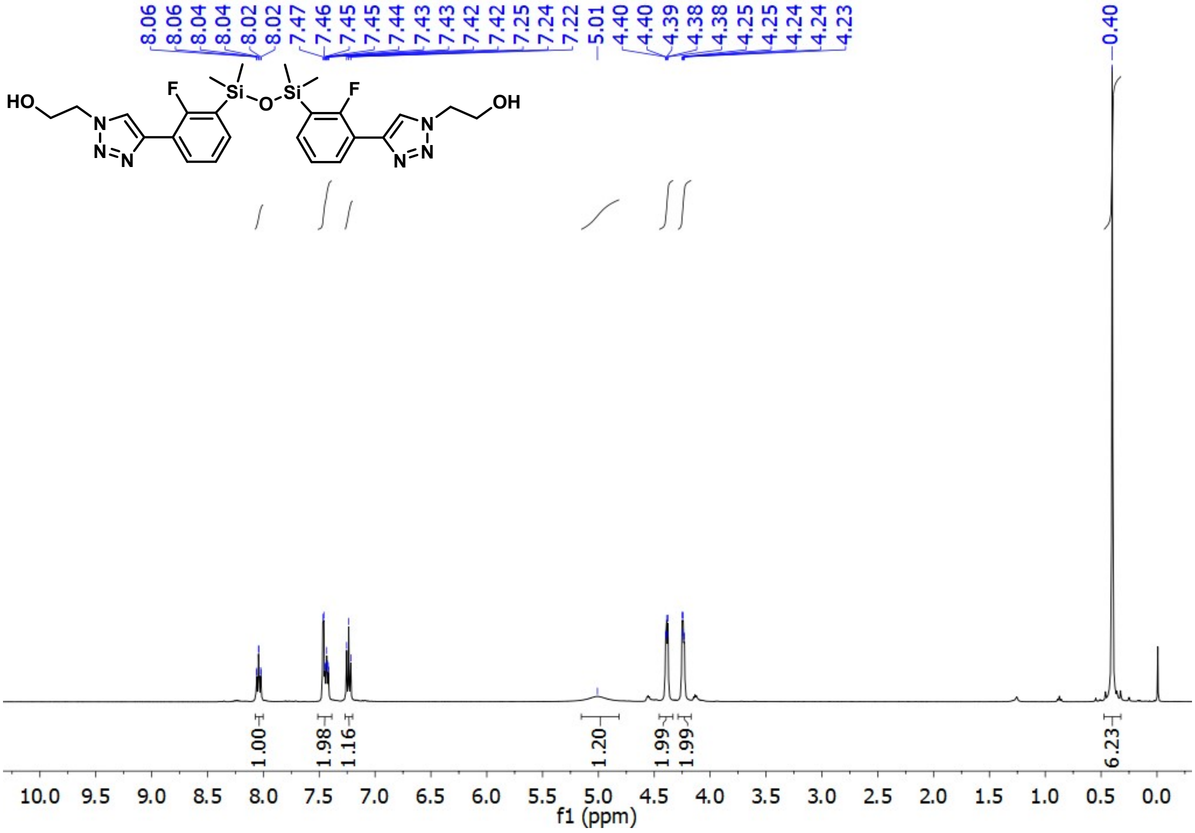

Figure S4.22. ^{1}H NMR (400 MHz, $CDCl_3$) spectrum of **5a**.

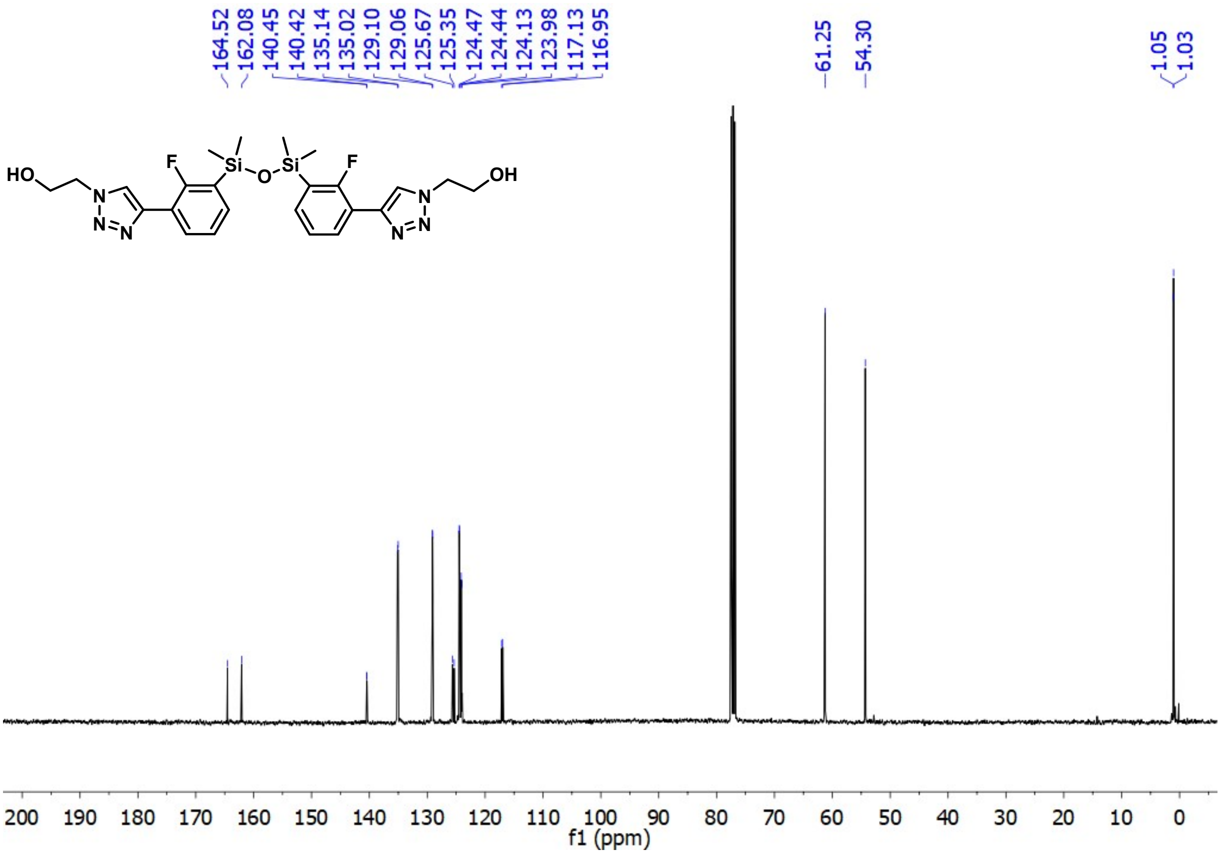

Figure S4.23. ^{13}C NMR (101 MHz, $CDCl_3$) spectrum of **5a**.

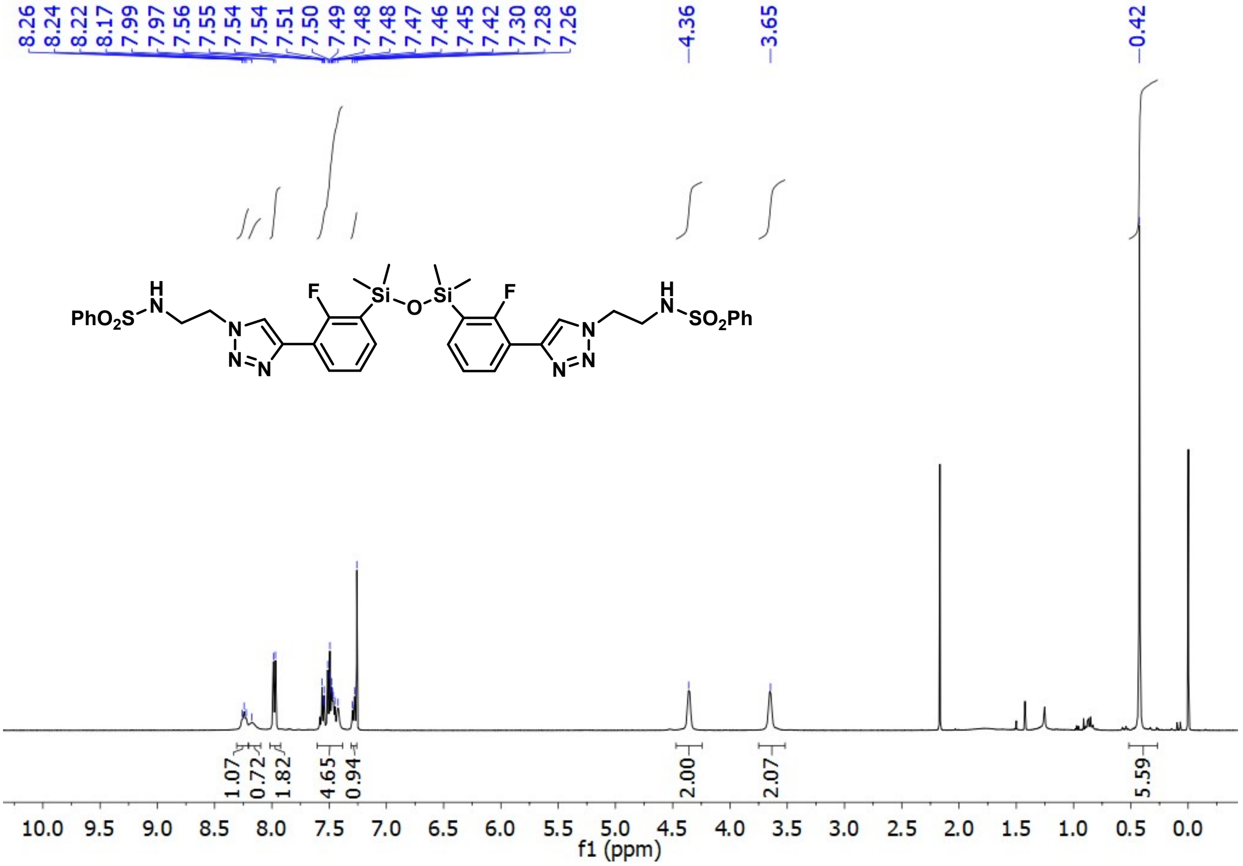

Figure S4.24. ^{1}H NMR (400 MHz, $CDCl_3$) spectrum of **5b**.

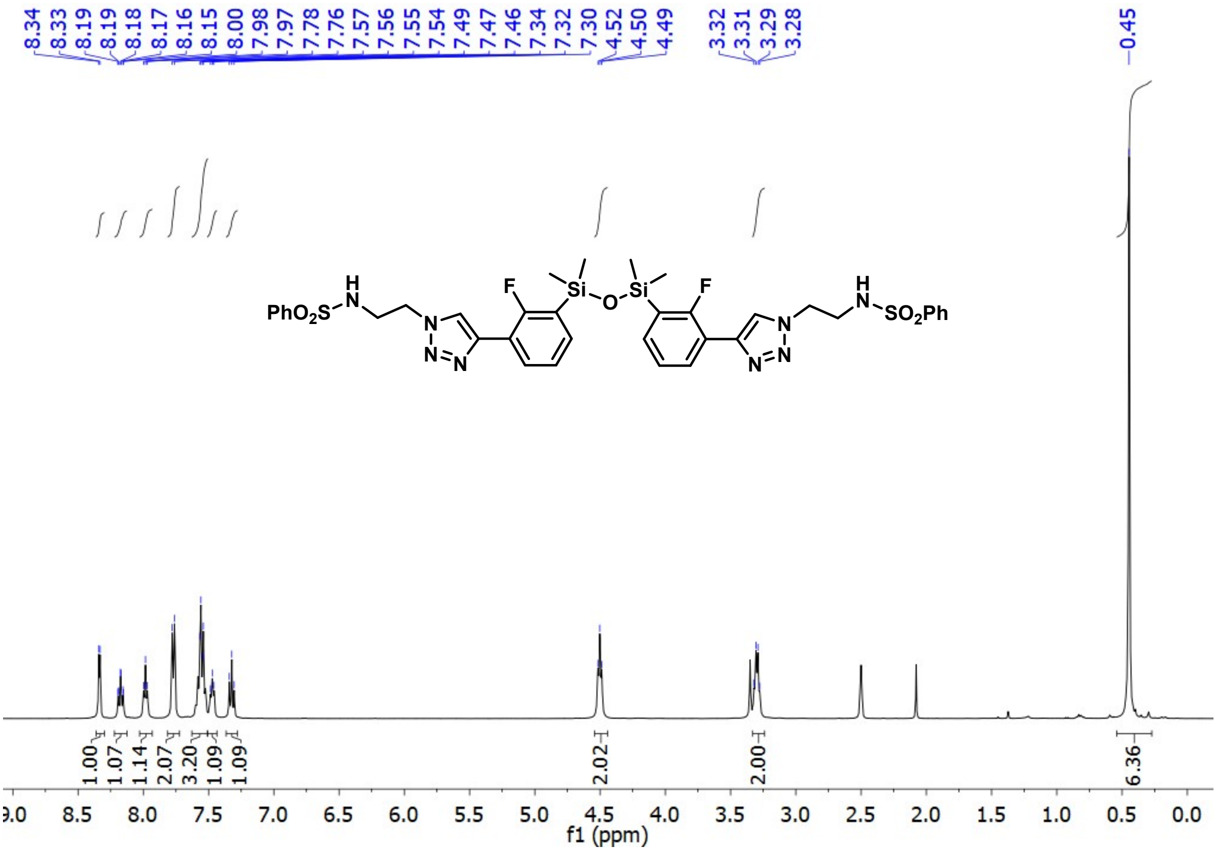

Figure S4.25. ^{1}H NMR (400 MHz, DMSO-d_6) spectrum of **5b**.

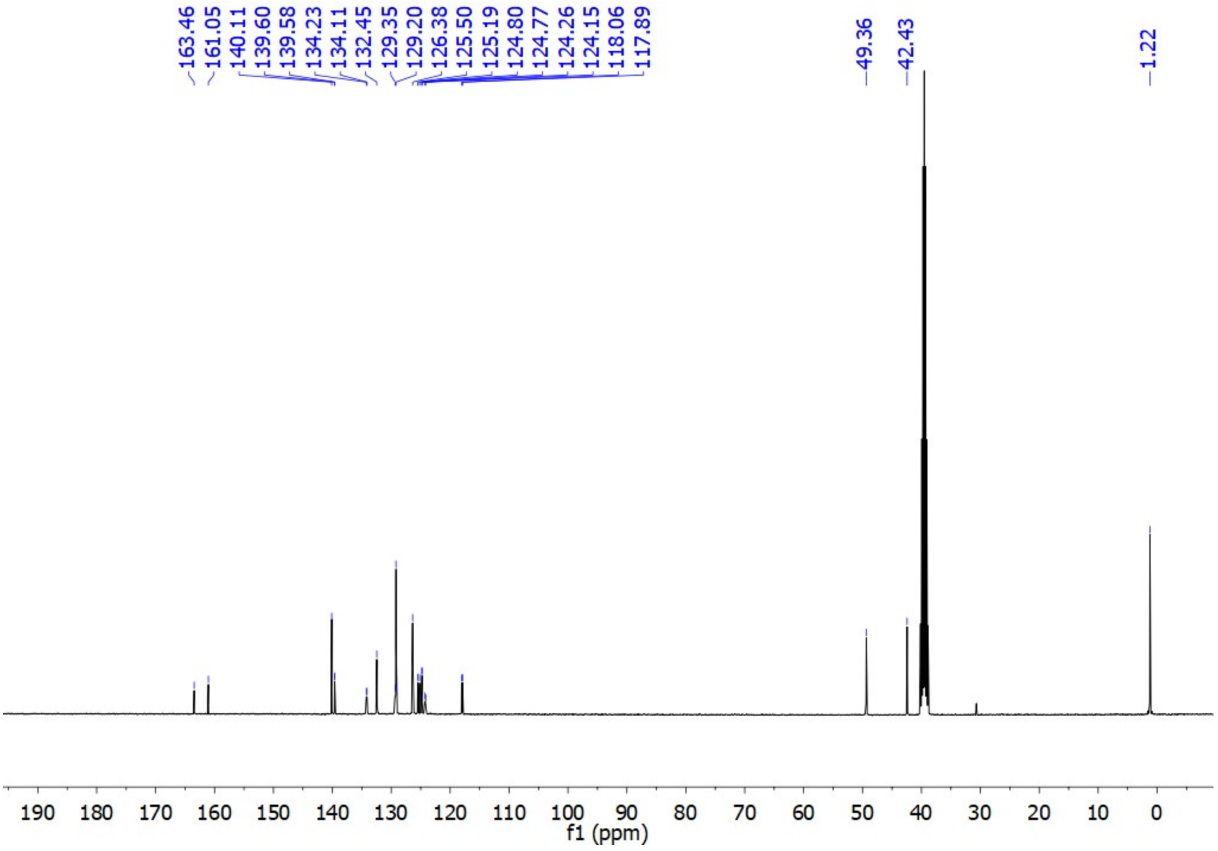

Figure S4.26. ^{13}C NMR (101 MHz, DMSO-d_6) spectrum of **5b**.
